# Multiple imputation in Cox regression when there are time‐varying effects of covariates

**DOI:** 10.1002/sim.7842

**Published:** 2018-07-16

**Authors:** Ruth H. Keogh, Tim P. Morris

**Affiliations:** ^1^ Department of Medical Statistics London School of Hygiene and Tropical Medicine London UK; ^2^ London Hub for Trials Methodology Research MRC Clinical Trials Unit at UCL, Aviation House London UK

**Keywords:** Cox regression, missing data, multiple imputation, restricted cubic spline, time‐varying effect

## Abstract

In Cox regression, it is important to test the proportional hazards assumption and sometimes of interest in itself to study time‐varying effects (TVEs) of covariates. TVEs can be investigated with log hazard ratios modelled as a function of time. Missing data on covariates are common and multiple imputation is a popular approach to handling this to avoid the potential bias and efficiency loss resulting from a “complete‐case” analysis. Two multiple imputation methods have been proposed for when the substantive model is a Cox proportional hazards regression: an approximate method (Imputing missing covariate values for the Cox model in Statistics in Medicine (2009) by White and Royston) and a substantive‐model‐compatible method (Multiple imputation of covariates by fully conditional specification: accommodating the substantive model in Statistical Methods in Medical Research (2015) by Bartlett et al). At present, neither accommodates TVEs of covariates. We extend them to do so for a general form for the TVEs and give specific details for TVEs modelled using restricted cubic splines. Simulation studies assess the performance of the methods under several underlying shapes for TVEs. Our proposed methods give approximately unbiased TVE estimates for binary covariates with missing data, but for continuous covariates, the substantive‐model‐compatible method performs better. The methods also give approximately correct type I errors in the test for proportional hazards when there is no TVE and gain power to detect TVEs relative to complete‐case analysis. Ignoring TVEs at the imputation stage results in biased TVE estimates, incorrect type I errors, and substantial loss of power in detecting TVEs. We also propose a multivariable TVE model selection algorithm. The methods are illustrated using data from the Rotterdam Breast Cancer Study. R code is provided.

## 1 INTRODUCTION

The setting of this paper is studies of associations between covariates and time‐to‐event outcomes such as disease diagnosis or death analysed using Cox regression.^1^, ^2^ Missing data in explanatory variables are common and the impacts of ignoring the missing data and performing a “complete‐case” analysis on the subset of individuals with no missing data are loss of efficiency and, depending on the missing data mechanism, biased estimates. Multiple imputation (MI) is a widely used approach for handling missing data that involves generating multiple plausible values for the missing data to create multiple imputed data sets. The multiply imputed data sets are each analysed to obtain estimates of interest and corresponding standard errors, which are then combined using rules developed by Rubin.^3^ The way in which the plausible values for missing data are obtained is important, and in general, use of a misspecified imputation model results in invalid inferences. In general, it is desirable that the imputation model is compatible with the chosen substantive model. There exists a range of methods for performing MI covering different substantive model types; see Carpenter and Kenward^4^ for an overview. Two MI approaches have been described for imputation of missing data on covariates in Cox regression. White and Royston^5^ outlined an approximately compatible method, which can be implemented in standard software, and Bartlett et al^6^ described an alternative “substantive‐model‐compatible” approach, which does not require approximations.

Assessment of whether the covariate effect changes over time is the basis of a test of the proportional hazards assumption, which is an important aspect of model assessment in Cox regression. Furthermore, in time‐to‐event analyses, it is often of interest to study whether the association of certain covariates with the hazard changes over time. Ignoring time‐varying effects (TVEs) and estimating an “average” hazard ratio can result in misleading conclusions.^7^ Cox^1^ described an extended version of his model to incorporate TVEs of covariates, and there is a large literature on methods for estimating and testing for TVEs in Cox regression: chapter 6 in Therneau and Grambsch^8^ summarises some of the more popular methods. There is also a more recent literature on model building in Cox regression incorporating TVEs.^9^, ^10^, ^11^, ^12^, ^13^

The existing imputation methods for handling missing data in Cox regression^5^, ^6^ do not account for TVEs of covariates, which could result in invalid inferences. In this paper, we extend the methods of White and Royston^5^ and Bartlett et al^6^ to accommodate imputation of covariates modelled with TVEs in the Cox regression model. The methods are presented for a general form for a TVE. Specific details are given for TVEs modelled using restricted cubic splines, which are flexible and do not require a form for the TVE to be prespecified. We also present a model selection algorithm that incorporates imputation of missing data into a procedure for testing for proportional hazards and selecting a flexible functional form for TVEs. Throughout, we make the assumption that data are “missing at random” (MAR).^3^, ^14^ Although the term “time‐varying effect” is used, we note that a hazard ratio changing over time does not necessarily correspond to a covariate's causal effect changing over time. It may instead occur when the association between a baseline covariate and the hazard becomes weaker (for example) over time or due to time‐varying confounding or omitted covariates (see Collett^15^ and Ford and Norrie^16^ for details).

The paper is organised as follows. In Section 2, we describe extensions to the methods of White and Royston^5^ and Bartlett et al^6^ to accommodate TVEs, for a general functional form for the TVEs. Use of restricted cubic splines to model the TVEs is described in Section 3. In Section 4, we discuss testing the proportional hazards assumption and present a model selection algorithm. The proposed methods are investigated using simulation studies described in Section 5, in which several underlying functional forms for the TVEs are considered. The methods are illustrated using data from the Rotterdam Breast Cancer Study in Section 6, and we conclude with a discussion in Section 7. R code for implementing the methods is available at https://github.com/ruthkeogh/MI-TVE.

## 2 MI FOR COX REGRESSION WITH TIME‐VARYING EFFECTS (TVEs)

### 2.1 Preliminaries

Let T denote the earlier of the event and censoring time and D be an indicator of whether an individual had the event (D=1) or was censored (D=0). For simplicity, we focus on a single covariate X_1_ with missing data and a fully observed covariate, X_2_. Extensions to missingness in several variables are described in the Supplementary Materials (Section S2). Under the extended Cox model that allows TVEs of covariates,^1^, ^8^ the hazard function can be written in the general form

(1) $$h(t|X_{1},X_{2})=h_{0}(t)\exp\left\{f_{X1}(t;\beta_{1})X_{1}+f_{X2}(t;\beta_{2})X_{2}\right\},$$

where h_0_(t) is the baseline hazard and the potential TVEs for X_1_ and X_2_ are represented respectively by the functions $f_{X1}(t;\beta_{1})$ and $f_{X2}(t;\beta_{2})$. Under the standard Cox proportional hazards model, ie, with no TVEs, $f_{X1}(t;\beta_{1})=\beta_{1}$ and $f_{X2}(t;\beta_{2})=\beta_{2}$.

### 2.2 MI overview

Using MI, the general procedure for obtaining estimates of the model parameters **β**_1_ and **β**_2_ is as follows (page 39 in the work of Carpenter and Kenward^4^). A model p(X_1_|T,D,X_2_;**α**) with noninformative prior on parameters **α** is specified for p(X_1_|T,D,X_2_), the distribution of X_1_ given T, D, and X_2_. Then, for m = 1,…,M,

1. The model p(X_1_|T,D,X_2_;**α**) is fitted by maximum likelihood to the data on individuals with observed X_1_ to obtain an approximate posterior distribution for **α**. A value **α** ^(m)^ is drawn is then drawn from this distribution.
2. For each individual i with missing X_1i_, a value $X_{1i}^{\left(m\right)}$ is drawn from p(X_1i_|T_i_,D_i_,X_2i_;**α** ^(m)^), giving an “imputed” data set in which there are no missing values.
3. The substantive model, here, the Cox regression model, is fitted to this imputed data set to give estimates $\left({\hat{\beta }}_{1}^{\left(m\right)},{\hat{\beta }}_{2}^{\left(m\right)}\right)$ of (**β**_1_,**β**_2_) and a corresponding estimate ${\hat{\Sigma }}^{\left(m\right)}$ of $\mathrm{Var}({\hat{\beta }}_{1}^{\left(m\right)},{\hat{\beta }}_{2}^{\left(m\right)})$.

Estimates $\left({\hat{\beta }}_{1}^{\left(m\right)},{\hat{\beta }}_{2}^{\left(m\right)}\right)$ (m = 1,…,M) and ${\hat{\Sigma }}^{\left(m\right)}$ are combined using “Rubin's rules” to give an overall estimate of (**β**_1_,**β**_2_) and corresponding variance‐covariance matrix.^3^

The main difficulty that arises when the substantive model is a Cox regression is that p(X_1_|T,D,X_2_) is a nonstandard distribution, which is only semiparametrized since h_0_(t) is nonparametric; therefore, we cannot easily draw values from the distribution p(X_1_|T,D,X_2_) to obtain the imputations. Although in principle any model p(X_1_|T,D,X_2_;**α**) could be used, potentially serious (asymptotic) bias in the estimates of (**β**_1_,**β**_2_) and their variance‐covariance matrix could arise if this model is misspecified. In particular, assuming the substantive model is correctly specified, if the imputation model is not compatible with the substantive model, under certain conditions, this implies that the imputation model is misspecified.^6^ Consequently, it is desirable that these two models be compatible (or approximately compatible), ie, that there exists a model for the joint distribution p(X_1_,T,D|X_2_) that implies as submodels the model p(X_1_|T,D,X_2_;**α**) used for imputation and the Cox model used for analysis. Two different approaches to using a compatible or approximately compatible imputation model have been described by Bartlett et al^6^ and White and Royston,^5^ which we refer to respectively as MI‐SMC and MI‐Approx. In the next two subsections, we describe extensions of these imputation methods to accommodate TVEs in the Cox regression model.

### 2.3 MI‐TVE‐Approx

For the standard Cox proportional hazards model assuming no TVEs ( f_X1_(t;**β**_1_) = β_1_ and f_X2_(t;**β**_2_) = β_2_), White and Royston^5^ showed that an approximately compatible imputation model for X_1_ is a logistic regression (for binary X_1_) or linear regression (for continuous X_1_) with linear predictor including main effects of D, X_2_, $\hat{H}(T)$ and the interaction between X_2_ and $\hat{H}(T)$, where $\hat{H}(T)$ is the Nelson‐Aalen estimate of the cumulative hazard. Investigations have found, in the settings examined, that the interaction term adds little.^5^, ^17^

When the substantive model is the extended Cox model with TVEs in 1, we can show that an approximately compatible imputation model for X_1_ is a logistic regression (for binary X_1_) or linear regression (for continuous X_1_) with linear predictor including main effects of X_2_, D f_X1_(T), $\hat{H}(T)$, ${\hat{H}}^{\left(1\right)}(T)$ and interactions of X_2_ with $\hat{H}(T)$ and ${\hat{H}}^{\left(1\right)}(T\;)$, where ${\hat{H}}^{\left(1\right)}(T\;)$ is the Nelson‐Aalen–type estimator ${\hat{H}}^{\left(1\right)}(T\;)=\sum_{t\leq T}\frac{td(t)}{n(t)}$ (d(t) and n(t) denote the number of events and number at risk at time t) and f_X1_(T ) denotes a vector of functions used for the TVE, eg if $f_{X1}$(t;**β**_1_)=β_10_+β_11_t then $f_{X1}(T)={\left(1,T\right)}^{\prime }$ . The details of the derivation are given in the Supplementary Materials (Section S1). We refer to the resulting approach as MI‐TVE‐Approx. In the simulations, we will investigate whether it is important to include the higher‐order cumulative hazard term ${\hat{H}}^{\left(1\right)}(T)$ and/or the interaction terms $X_{2}\times \hat{H}(T)$ and $X_{2}\times {\hat{H}}^{\left(1\right)}(T)$. The imputation procedure is as follows.

1. Fit the imputation model to the subset of individuals with complete data. For continuous X_1_, this is
  $$X=\alpha_{0}+\alpha_{1}X_{2}+\alpha_{2}^{\prime }Df_{X1}(T\;)+\alpha_{3}\hat{H}(T\;)+\alpha_{4}{\hat{H}}^{\left(1\right)}(T\;)+\alpha_{5}X_{2}\hat{H}(T\;)+\alpha_{6}X_{2}{\hat{H}}^{\left(1\right)}(T\;)+\varepsilon ,$$
  and for binary X_1_,
  $$\text{logit}\;\mathrm{Pr}(X_{1}=1|T,D,X_{2})=\alpha_{0}+\alpha_{1}X_{2}+\alpha_{2}^{\prime }Df_{X1}(T\;)+\alpha_{3}\hat{H}(T\;)+\alpha_{4}{\hat{H}}^{\left(1\right)}(T\;)+\alpha_{5}X_{2}\hat{H}(T\;)+\alpha_{6}X_{2}{\hat{H}}^{\left(1\right)}(T\;).$$
2. Take M random draws values of the parameters from their approximate posterior distribution (we refer to the work of White et al^18^ for details), denoted $\alpha_{j}^{\left(m\right)}$ ( j = 0,1…,6)(binary and continuous X_1_), and additionally $\sigma_{\varepsilon }^{2(m)}$ for continuous X_1_, where $\sigma_{\varepsilon }^{2}$ denotes the variance of the residuals ε.
3. The imputed value of X_1i_ in the mth imputed data set is given (for continuous X_1_) by
  $$X_{1i}^{\left(m\right)}=\alpha_{0}^{\left(m\right)}+\alpha_{1}^{\left(m\right)}X_{2}+\alpha_{2}^{{\left(m\right)}^{\prime }}Df_{X1}(T)+\alpha_{3}^{\left(m\right)}\hat{H}(T)+\alpha_{4}^{\left(m\right)}{\hat{H}}^{\left(1\right)}(T)+\alpha_{5}^{\left(m\right)}X_{2}\hat{H}(T)+\alpha_{6}^{\left(m\right)}X_{2}{\hat{H}}^{\left(1\right)}(T)+\varepsilon^{\left(m\right)},$$
  where ε ^(m)^ is a random draw from a normal distribution with mean 0 and variance $\sigma_{\varepsilon }^{2(m)}$. For binary X_1_, the imputed value is a draw from a Bernoulli distribution with
  $$\text{logit}\;\mathrm{Pr}(X_{1}=1|T,D,X_{2})=\alpha_{0}^{\left(m\right)}+\alpha_{1}^{\left(m\right)}X_{2}+\alpha_{2}^{{\left(m\right)}^{\;\prime }}Df_{X1}(T\;)+\alpha_{3}^{\left(m\right)}\hat{H}(T\;)+\alpha_{4}^{\left(m\right)}{\hat{H}}^{\left(1\right)}(T\;)+\alpha_{5}^{\left(m\right)}X_{2}\hat{H}(T\;)+\alpha_{6}^{\left(m\right)}X_{2}{\hat{H}}^{\left(1\right)}(T\;).$$

### 2.4 MI‐TVE‐SMC

In the context of the standard Cox proportional hazards model without TVEs, MI‐Approx has been found to work well in a range of circumstances.^4^, ^5^ However, the approximation can perform badly in some “extreme” situations, including when there are large effect sizes and when the event rate is high.^5^ Bartlett et al^6^ described an approach, referred to here as MI‐SMC, which ensures that the imputation model is compatible with the user's chosen substantive model; here, a Cox regression (“substantive‐model‐compatible” – SMC). However, they did not accommodate TVEs.

To extend the method of Bartlett et al^6^ to modelling of TVEs, we adapt the approach first proposed in Bartlett's PhD thesis^19^ for handling missing data in time‐dependent covariates, noting the close connection between time‐dependent covariates and TVEs. We refer to the resulting method as MI‐TVE‐SMC. The MI‐TVE‐SMC imputation procedure is as follows.

First, a model p(X_1_|X_2_;**γ**) is specified. For binary X_1_, this may be a logistic regression model, and for continuous X_1_, a linear regression model. The steps used to obtain the mth imputed data set are then as follows.

1. Fill in the missing variables with arbitrary starting values to create a complete data set.
2. Fit the Cox regression model of interest, including TVEs, to the current complete data set to obtain estimates $\left({\hat{\beta }}_{1},{\hat{\beta }}_{2}\right)$ and their estimated variance‐covariance matrix $\hat{\Sigma }$. Draw values $\beta_{1}^{\left(m\right)},\beta_{2}^{\left(m\right)}$ from a joint normal distribution with mean $\left({\hat{\beta }}_{1},{\hat{\beta }}_{2}\right)$ and variance‐covariance matrix $\hat{\Sigma }$.
3. Calculate Breslow's estimate,^20^ denoted $H_{0}^{\left(m\right)}(t)$, of the baseline cumulative hazard H_0_(t) using parameter values $\beta_{1}^{\left(m\right)},\beta_{2}^{\left(m\right)}$ and current imputations of X_1_.
4. Estimate parameters **γ** and their variance‐covariance by fitting the assumed regression model for X_1_ on X_2_ to the current complete data set. Draw a value $\gamma^{*}$ from the approximate joint posterior distribution of **γ**.^18^
5. For each individual for whom X_1_ is missing, (i) draw a value $X_{1}^{*}$ from the distribution $p\;(X_{1}|X_{2};\gamma^{*})$ and (ii) draw a value U from a uniform distribution on [0,1]. Accept the value $X_{1}^{*}$ if
  $$\left\{\begin{array}{cc} U\leq \exp\left[-\sum_{j:t_{j}\leq T}\Delta H_{0}^{\left(m\right)}(t_{j})\exp\left\{f_{X1}\left(t_{j};\beta_{1}^{\left(m\right)}\right)X_{1}^{*}+f_{X2}\left(t_{j};\beta_{2}^{\left(m\right)}\right)X_{2}\right\}\right] & \text{if}\;D=0\\ U\leq \Delta H_{0}^{\left(m\right)}(T)\exp\left\{1+f_{X1}\left(T;\beta_{1}^{\left(m\right)}\right)X_{1}^{*}+f_{X2}\left(T;\beta_{2}^{\left(m\right)}\right)X_{2}\right.\\ \left.-\sum_{j:t_{j}\leq T}\Delta H_{0}^{\left(m\right)}(t_{j})e^{f_{X1}\left(t_{j};\beta_{1}^{\left(m\right)}\right)X_{1}^{*}+f_{X2}\left(t_{j};\beta_{2}^{\left(m\right)}\right)X_{2}}\right\} & \text{if}\;D=1, \end{array}\right.$$
  where $\Delta H_{0}^{\left(m\right)}(t)$ denotes the increment in $H_{0}^{\left(m\right)}(t)$ at time t and t_1_,..,t_k_ denote the unique event times. Repeat (i) and (ii) until a value $X_{1}^{*}$ is accepted.
6. Return to steps 2–5 until the imputed X_1_ values have converged to a stationary distribution. These are then the imputed values in the mth imputed data set.

The difference between the MI‐SMC approach, which does not accommodate TVEs, and the MI‐TVE‐SMC approach is in the terms used for the rejection in step 5 and the fact that a Cox model with TVEs is fitted in Step 2.

We have outlined the MI‐TVE‐Approx and MI‐TVE‐SMC approaches for the simple setting of missing data in a single covariate X_1_ with a TVE. Both methods extend to handle missingness in several covariates using the fully conditional specification approach (also referred to as multiple imputation by chained equations), in which an imputation model is specified for each partially missing covariate conditional on all the other covariates and an iterative approach is used to fit the imputation models.^18^, ^21^ Details are provided in the Supplementary Materials (Section S2).

## 3 FUNCTIONAL FORM OF TIME‐VARYING EFFECTS (TVEs)

In the preceding section, the MI methods were described for a general functional form for the TVEs, f_X_(t;**β**). Approaches to modelling TVEs include use of prespecified functional forms^22^ (eg, f_X_(t;**β**)=β_0_+β_1_ t), step‐functions,^22^, ^23^, ^24^ fractional polynomials,^12^ and splines.^10^, ^11^, ^25^, ^26^, ^27^, ^28^, ^29^, ^30^ In this paper, we focus on using a restricted cubic spline form^25^, ^26^ for the TVE function because this allows a flexible form for the TVE with relatively few parameters. Under a restricted cubic spline with L knots at u_1_,…,u_L_, the TVE function for a covariate X is

(2) $$f_{X}(t;\beta )=\beta_{0}+\beta_{1}t+\sum_{i=1}^{L-2}\theta_{i}\left\{{\left(t-u_{i}\right)}_{+}^{3}-\left(\frac{{\left(t-u_{L-1}\right)}_{+}^{3}(u_{L}-u_{i})}{\left(u_{L}-u_{L-1}\right)}\right)+\left(\frac{{\left(t-u_{L}\right)}_{+}^{3}(u_{L-1}-u_{i})}{\left(u_{L}-u_{L-1}\right)}\right)\right\},$$

where (t−u_i_)_+_ takes value (t−u_i_) if (t−u_i_)>0 and 0 otherwise, and ***β*** includes β_0_, β_1_ and θ_i_(i=1,…,L−2). The number of knots used and the position of the knots have to be decided by the user, and there is no formal theoretical basis for the decision. Hess^25^ noted empirical evidence that three to five knots are usually adequate and the fit is not greatly altered by altering the knot positions. Stone^31^ also recommended using five knots in restricted cubic splines. Hess^25^ suggested placing knots at quantiles of the observed follow‐up times (including both event times and censoring times), with the outer knots near the extremes, and the internal knots placed approximately uniformly over the quantiles of the distribution of the follow‐up times. Similar recommendations were given by Durrleman and Simon^32^ in the context of restricted cubic splines for functional forms of covariates in survival analyses. In the simulations, we consider using restricted cubic splines with five knots placed at percentiles (5th, 25th, 50th, 75th, 95th) of the event time distribution (ie, excluding censoring times).

In the case of a restricted cubic spline with L=5 knots, the MI‐TVE‐Approx imputation model for X_1_ should include X_2_, D, the interaction between D and T, interactions of D with $\left\{{\left(T-u_{i}\right)}_{+}^{3}-\frac{{\left(T-u_{L-1}\right)}_{+}^{3}(u_{L}-u_{i})}{\left(u_{L}-u_{L-1}\right)}+\frac{{\left(T-u_{L}\right)}_{+}^{3}(u_{L-1}-u_{i})}{\left(u_{L}-u_{L-1}\right)}\right\}$ for i = 1,2,3, $\hat{H}(T\;)$, ${\hat{H}}^{\left(1\right)}(T\;)$, and interactions of X_2_ with $\hat{H}(T\;)$ and ${\hat{H}}^{\left(1\right)}(T\;)$.

## 4 TESTING THE PROPORTIONAL HAZARDS ASSUMPTION AND MODEL SELECTION

In most contexts, when using Cox regression modelling, it is important to assess whether covariates have TVEs, that is, to perform tests of the proportional hazards assumption. TVEs can then be included for covariates for which the proportional hazards assumption appears not to hold. Tests of proportional hazards based on TVEs modelled using splines have been previously described by Abrahamowicz et al.^27^ With fully observed data on covariates, the proportional hazards assumption can be tested using a likelihood ratio test, comparing a model including TVEs to the model without TVEs. A joint Wald test of the TVE parameters is asymptotically equivalent: assuming a restricted cubic spline for the TVE for X (Equation 2), this is a joint test of β_1_=θ_1_=⋯=θ_L−2_=0. Wood et al^33^ described the use of Wald tests for model selection using multiply imputed data, and this was further evaluated by Morris et al^34^ in the context of covariate transformations based on fractional polynomials. We suggest this approach for tests of TVEs. The joint test of the parameters of interest (null hypothesis β_1_=θ_1_=⋯=θ_L−2_=0) uses the pooled parameter estimates and the corresponding variance covariance matrix obtained from Rubin's rules. We use the Wald‐type test described by Li et al^35^ and Meng and Rubin^36^ as this was found to perform slightly better in terms of giving correct type I errors in subsequent simulation studies (Section 5).

For the purposes of testing the proportional hazards assumption as part of a model assessment and selection procedure, we recommend allowing TVEs for all variables at the imputation stage of the analysis; the importance of doing so for valid tests of the proportional hazards assumption is investigated in the simulation studies.

Several authors have proposed algorithms for model selection involving both TVEs and transformation of covariates,^10^, ^11^, ^12^ though all assume fully observed data. We propose an algorithm (the MI‐MTVE algorithm), which provides a model selection procedure for identifying TVEs using multiply imputed data. The MI‐MTVE algorithm is an adaptation of the “multivariable fractional polynomial‐time” (MFPT) algorithm of Sauerbrei et al,^12^ which uses fractional polynomial transformations of covariates and fractional polynomial forms for TVEs. Our adaptation employs restricted cubic splines rather than fractional polynomials for TVEs, and is similar to a procedure advocated by Wynant and Abrahamowicz.^11^ Forward selection is used to accommodate investigation of TVEs in multiple covariates and selection of functional forms for TVEs using restricted cubic splines with up to five knots.

**MI‐MTVE algorithm**

Step 1: Perform MI‐TVE‐Approx or MI‐TVE‐SMC, assuming a restricted cubic spline with five knots for the TVE for each covariate to obtain M imputed data sets.
Step 2: In each imputed data set, fit the Cox regression model with no TVEs of any covariate (denoted model $M_{0}$). Denote the set of covariates by C. For each c (c ∈ C) and in each imputed data set, fit four Cox regression models including TVEs for covariate c of increasing complexity (indexed by j ): using the linear form ( f_X_(t;**β**) = β_0_ + β_1_ t) and restricted cubic splines with three, four, and five knots. The remaining covariates in the Cox regression are not modelled using TVEs.
Step 3: For each covariate c, perform a test for TVEs in each model j using joint Wald tests for multiply imputed data, as described above.^35^, ^36^ Select the combination of covariate (c) and TVE model ( j ) that returns the smallest p‐value in the test for TVEs. If no combination of c and j gives a p‐value less than a chosen level α, stop; the working model without TVEs $M_{0}$ is adequate. Otherwise, update the working model $M_{0}$ to include TVEs for the covariate c using the TVE model j that returned the smallest p‐value. Call this new working model $M_{1}$.
Step 4: Repeat steps 2–3 with updated working models until there are no remaining covariates c not in the current working model that have a significant TVE (at level α) under any TVE model j. Stop; this working model is the final selected model.

The estimates of the parameters of the final selected model and corresponding estimated variance‐covariance matrix are those obtained by applying Rubin's rules to the results from fitting the final model to each imputed data set. The MI is performed only in step 1 and is based on a TVE for each covariate of the most complex form that we consider in this paper (a restricted cubic spline with five knots). This means that a restriction of the imputation model should be compatible with the model selected by the algorithm (termed “semi‐compatibility”).^6^, ^34^, ^37^ The imputation model may include some redundant parameters, but this will not impact on the validity of MI inference. Collins et al^38^ showed that the cost in terms of efficiency of including many predictors in the imputation model (ie, with potentially many redundant parameters) is relatively small and that this is outweighed by the benefits in terms of potential to avoid bias and actually gain efficiency.

## 5 SIMULATION STUDY

We now present a simulation study which was designed to evaluate the MI methods across a variety of data‐generating scenarios.

### 5.1 Data‐generating mechanisms

Data were generated for a cohort of 2000 individuals. Two covariates, X_1_ and X_2_, are considered. Event times T_E_ were generated according to the exponential hazard model

(3) $$h(t|X_{1},X_{2})=\lambda_{E}\exp\left\{f_{X1}(t;\beta_{1})X_{1}+f_{X2}(t;\beta_{2})X_{2}\right\}.$$

**Figure 1:**
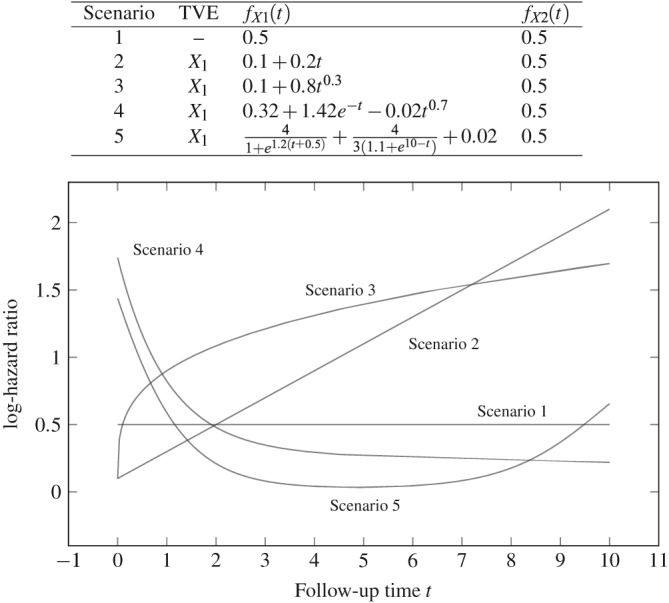
Time‐varying effect (TVE) functions used in simulation studies

We consider five forms for the TVEs. These are listed in the table at the top of Figure 1. In scenario 1, neither covariate has a TVE. In scenarios 2–5, X_1_ has a TVE but X_2_ does not. Figure 1 depicts the form of the TVEs. These forms include examples previously used by Buchholz^39^ and Buchholz and Sauerbrei.^9^

Random drop out times, *T*_*C*_, were generated according to an exponential distribution with rate *λ*_*C*_, and administrative censoring was imposed after 10 years of follow‐up. The observed time for each individual was calculated as *T* = min(*T*_*E*_,*T*_*C*_,10). Values for *λ*_*E*_ and *λ*_*C*_ were chosen such that 10% of individuals have the event of interest and 50% are censored because of random drop out, with the remainder being administratively censored.

Both binary and continuous pairs of covariates are considered. Binary *X*_1_ was generated from a binomial distribution such that *P*(*X*_1_=1)=0.2, and binary *X*_2_ was generated using logit{*P*(*X*_2_=1|*X*_1_)}=*X*_1_. Continuous *X*_1_ and *X*_2_ were generated from a bivariate normal distribution with means 0, variances 1, and correlation 0.5.

Nonmonotone missing data were generated in *X*_1_ and *X*_2_ according to a MAR mechanism in which the probability of missingness in *X*_1_ depends on observed values of *X*_2_ and vice versa (see Supplementary Materials Section S4). In this, *X*_1_ is missing for 30% of individuals and *X*_2_ for 30% of individuals, resulting in approximately 50% of individuals missing at least one of the measurements.

There are 10 main simulation scenarios: five different scenarios for TVEs of *X*_1_ and binary and continuous *X*_1_ and *X*_2_. Five hundred simulated data sets were generated under each scenario (justified in the Supplementary Materials Section S5). In Section 5.5, we present results from additional sensitivity scenarios with a higher event rate, lower level of missingness, and a MAR mechanism in which the missingness in *X*_1_ and *X*_2_ additionally depends on the outcome *D*.

### 5.2 Methods compared

The methods we investigate are the proposed MI‐TVE‐Approx and MI‐TVE‐SMC approaches, and for comparison, the corresponding approaches that do not incorporate TVEs (MI‐Approx and MI‐SMC). We also performed a complete‐data analysis (before missing data is introduced) and a complete‐case analysis, which uses only the subset with no missing data. In MI‐Approx, we omitted the interaction terms between covariates and $\hat{H}(T)$ because their inclusion was not found to result in material differences in the results. For the same reason, in MI‐TVE‐Approx, we omitted the interaction terms and terms involving ${\hat{H}}^{\left(1\right)}(T)$. The MI‐TVE‐Approx imputation model recommended for *X*_1_ therefore includes *X*_2_, *D*, the interaction between *D* and *T*, interactions of *D* with $\left\{{\left(T-u_{i}\right)}_{+}^{3}-\frac{{\left(T-u_{L-1}\right)}_{+}^{3}(u_{L}-u_{i})}{\left(u_{L}-u_{L-1}\right)}+\frac{{\left(T-u_{L}\right)}_{+}^{3}(u_{L-1}-u_{i})}{\left(u_{L}-u_{L-1}\right)}\right\}$ for *i* = 1,2,3, and $\hat{H}(T)$. The recommended imputation model for *X*_2_ is the same but with *X*_2_ replaced by *X*_1_.

In all Cox regression analyses, TVEs for *X*_1_ and *X*_2_ are modelled using a restricted cubic spline with five knots placed at percentiles (5th, 25th, 50th, 75th, 95th) of the distribution of the observed event times. This includes a TVE model for *X*_2_( *f*_*X*2_(*t*;***β***_2_)) even though, in the data‐generating process, there is no TVE of *X*_2_. In MI‐TVE‐Approx and MI‐TVE‐SMC, the TVE was incorporated on the basis of the same functional form.

In the MI analyses, we used 10 imputed data sets. For the analysis of studies in practice, we recommend the rule of thumb suggested by White et al^18^ to set the number of imputations to be approximately the same as the percentage of missing data, with a larger number chosen if numerical reproducibility of estimates is desired.

### 5.3 Performance measures

The performance of methods was assessed in a number of ways as described below. Each assessment was performed for both *X*_1_ and *X*_2_.

- Curvewise estimate of the TVE, presented visually over the follow‐up time and averaged over simulation runs.
- Bias in the estimated curve at one, five, and nine years and corresponding 95% Monte Carlo confidence intervals. The bias from the MI methods and the complete‐case analysis was calculated relative to the complete‐data results, ie, as the difference between the MI or complete‐case estimates and the mean of the complete‐data estimates. This was done because the true data‐generating mechanism is not a restricted cubic spline and therefore we do not necessarily expect to get completely unbiased estimates from the complete‐data analysis.
- Coverage of confidence intervals, estimated at one, five, and nine years, defined as the proportion of simulated data sets for which the true curve lies within the 95% confidence intervals at time *t*=1,5,9.
- Curvewise root mean squared error (RMSE), presented visually over the follow‐up time and averaged over simulation runs.
- Rejection fractions for the test of the proportional hazards assumption. For scenario 1, in which there are no TVEs, this corresponds to a type I error rate for the TVEs of both *X*_1_ and *X*_2_. For all other scenarios, this corresponds to power for the TVE of *X*_1_ and type I error rate for the TVE of *X*_2_. The proportional hazards assumption is assessed using a joint Wald test of the TVE parameters (see Section 4).

All simulations and analyses were performed using R. The substantive model was fitted using coxph in the survival package. MI‐Approx and MI‐TVE‐Approx were implemented using mice 40 and MI‐SMC using smcfcs (https://github.com/jwb133/smcfcs). We extended the smcfcs code to implement MI‐TVE‐SMC in R, and this is available at https://github.com/ruthkeogh/MI-TVE, together with simulation examples. The code allows the user to specify the TVE function.

### 5.4 Simulation results

#### 5.4.1 Bias, coverage, and RMSE

**Figure 2:**
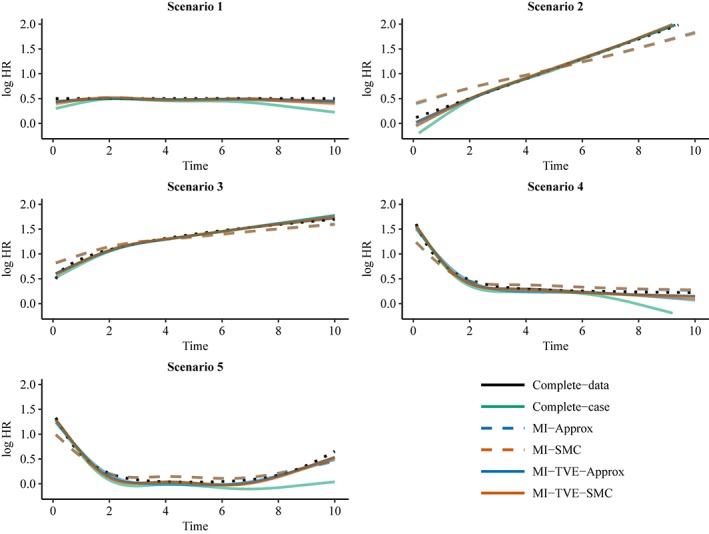
Curve‐wise estimates of TVEs for covariate X_1_ in the setting with binary covariates X_1_ and X_2_. The dotted black line indicates the true curve. MI, multiple imputation; SMC, substantive‐model‐compatible; TVE, time‐varying effect [Colour figure can be viewed at http://wileyonlinelibrary.com]

**Figure 3:**
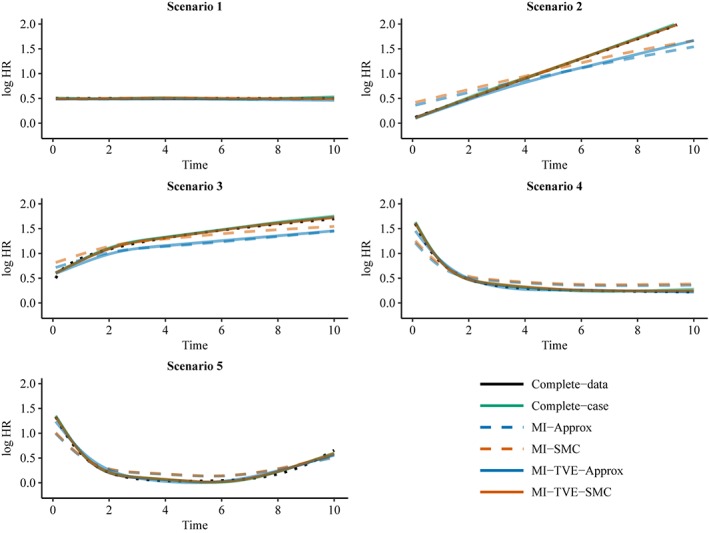
Curve‐wise estimates of TVEs for covariate X_1_ in the setting with continuous covariates X_1_ and X_2_. The dotted black line indicates the true curve. MI, multiple imputation; SMC, substantive‐model‐compatible; TVE, time‐varying effect [Colour figure can be viewed at http://wileyonlinelibrary.com]

Figures 2 and 3 show the curvewise TVE estimates for covariate *X*_1_ in the binary and continuous covariates settings. Supplementary Figures S1 and S2 show the bias in the estimated TVE curves at three time points (1, 5, 9), corresponding to the difference between the mean curves shown in Figures 2 and 3 and the true curve or that obtained from the complete data analysis. Similar plots for *X*_2_, which always has a time‐constant effect, are shown in the Supplementary Materials (Figures S3 and S4).

The complete‐data and complete‐case analyses give approximately unbiased TVE estimates except for some bias in the complete‐case analysis in the extremes of some curves. As noted earlier, the complete‐data analysis could give estimates with some slight bias because the data were not generated under the restricted cubic spline model, which is used in the analysis. Note that we expect the complete‐case analysis to give an approximately unbiased result because the missingness does not depend on the outcome. The results from MI‐Approx and MI‐SMC show that failing to account for the TVE in the imputation gives a biased estimate of the TVE curve. The bias is such that the TVE curve appears more flat and the HR is overestimated at some times and underestimated at others. For example, in Scenario 2, where the true TVE is linear, the slopes of the estimated TVE from MI‐Approx and MI‐SMC are less steep than the true slope. The MI methods that accommodate TVEs, MI‐TVE‐Approx and MI‐TVE‐SMC, perform similarly for binary *X*_1_ and give TVE estimates similar to that from the complete‐data analysis. However, for continuous *X*_1_, only MI‐TVE‐SMC performs well in general, with MI‐TVE‐Approx giving clearly biased estimates in scenarios 2 and 3 at times where the TVE is quite large. MI‐TVE‐Approx requires additional approximations for continuous covariates, and the approximation does not perform well in these scenarios. Poor performance of MI‐Approx in scenarios with continuous covariates and large effect sizes has been found previously in the setting without TVEs.^5^

Tables of coverages of the estimated TVE curves at three time points are given in Supplementary Tables S1 and S2. The coverage levels tend to be higher than the nominal 95% level at the later timepoints, with many reaching 100%. This is not an issue specific to MI since we see the same behaviour for the analysis of complete data; it is at least reassuring that coverage results for the MI‐TVE‐SMC method follow a very similar pattern to those from the complete‐data analysis. Coverage not at the nominal level has been previously observed for spline‐based models.^41^

Supplementary Figures S5 and S6 show curvewise RMSE. As we expect, the complete‐case analysis gives the largest RMSE. The RMSEs from MI‐TVE‐Approx and MI‐TVE‐SMC are slightly larger than those from the complete‐data analysis and from the MI analyses that ignore TVEs. However, these plots show that there is not a substantial bias‐variance trade‐off in the MI analyses that accommodate TVEs. Supplementary Figures S7 and S8 further illustrate this point by showing estimated curves from 100 example simulated data sets using the complete‐data analysis and MI‐TVE‐SMC: the curves obtained using MI‐TVE‐SMC have a greater spread than those from the complete‐data analysis, as we would expect, but not greatly so.

#### 5.4.2 Tests of the proportional hazards assumption

Table 1 shows the percentage of simulations in which the proportional hazards assumption (based on joint Wald tests) was rejected at the 5% level for *X*_1_ and *X*_2_ in the binary and continuous covariates settings. In scenario 1, where neither *X*_1_ nor *X*_2_ has a TVE, the percentage of simulations in which the null hypothesis of proportional hazards was rejected was close to 5% in the complete‐data and complete‐case analyses, and also in MI‐TVE‐Approx and MI‐TVE‐SMC, indicating approximately correct type I errors. However, the results of zero from MI‐Approx and MI‐SMC show that failing to account for the possibility of TVEs in the imputation model will result in incorrect type I errors. Type I error results are similar for *X*_2_, which has a time‐constant effect across scenarios. In scenarios 2–5 with TVEs for *X*_1_, the power to reject the null hypothesis of proportional hazards varied under the complete‐data analysis, from 100% (continuous covariates, scenario 2) to 33% (binary covariates, scenario 3). Power was generally lower in the setting with binary covariates. Power was reduced under the complete‐case analysis; for example, in scenario 4 with continuous covariates, the power from the complete‐case analysis was 78% compared with 99% in the complete‐data analysis, and in scenario 4 with binary covariates, the power from the complete‐case analysis was 17% compared with 56% in the complete‐data analysis. For binary covariates, the power under MI‐TVE‐Approx and MI‐TVE‐SMC was increased relative to the complete‐case analysis and was highest for MI‐TVE SMC. Power using MI‐TVE‐SMC was also high in the setting with continuous covariates but lower for MI‐TVE‐Approx and, in scenarios 2 and 3, lower than that from the complete‐case analysis; this is partly a consequence of the bias observed using MI‐TVE‐Approx for continuous covariates. The results show that if the TVEs are ignored in the imputation (MI‐Approx and MI‐SMC), there is a large loss of power to reject the null hypothesis of proportional hazards across all scenarios, and power from these methods was considerably lower than that from the complete‐case analysis.

**Table 1:** Percentage of simulations in which the null hypotheses of proportional hazards for X_1_ and X_2_ were rejected using joint Wald tests^35^, ^36^ for binary and continuous covariates. For a given rejection percentage π, the Monte Carlo SE is $\sqrt{\frac{\pi (100-\pi )}{500}\times \frac{1}{100}}$

	Scenario 1		Scenario 2		Scenario 3		Scenario 4		Scenario 5	
	*X*_1_	*X*_2_	*X*_1_	*X*_2_	*X*_1_	*X*_2_	*X*_1_	*X*_2_	*X*_1_	*X*_2_
*Binary* *X*1,*X*2										
Complete‐data	3	3	89	3	33	3	56	6	45	4
Complete‐case	2	3	42	3	14	2	17	2	14	3
MI‐Approx	0	0	21	0	3	0	4	0	2	0
MI‐SMC	0	0	17	0	2	0	3	0	1	0
MI‐TVE‐Approx	2	3	67	3	16	2	27	2	21	2
MI‐TVE‐SMC	3	4	68	6	24	6	34	5	27	5
*Continuous* *X*1,*X*2										
Complete‐data	7	5	100	3	78	5	99	3	96	4
Complete‐case	5	6	94	5	42	4	78	4	60	5
MI‐Approx	0	0	70	0	7	0	40	0	28	0
MI‐SMC	0	0	82	0	9	0	48	0	30	0
MI‐TVE‐Approx	4	5	91	2	23	2	83	3	70	3
MI‐TVE‐SMC	5	7	99	4	53	5	88	3	75	5

Abbreviations: MI, multiple imputation; SMC, substantive‐model‐compatible; TVE, time‐varying effect.

### 5.5 Additional simulation investigations

We investigated the performance of the methods in three additional situations.

- Missingness depends on the outcome (see Supplementary Materials Section S4). Missingness depending on the outcome is plausible if there is an underlying latent feature that is associated with the subsequent outcome and with missingness.
- Fifty percent of individuals have the event. This may not be a common situation but is relevant for certain clinical studies.
- A lower percentage of individuals with missing covariate data. The percentage of individuals missing *X*_1_ and missing *X*_2_ was reduced to 10% (see Supplementary Materials Section S4), which results in approximately 20% of individuals missing at least one of the measurements.

**Figure 4:**
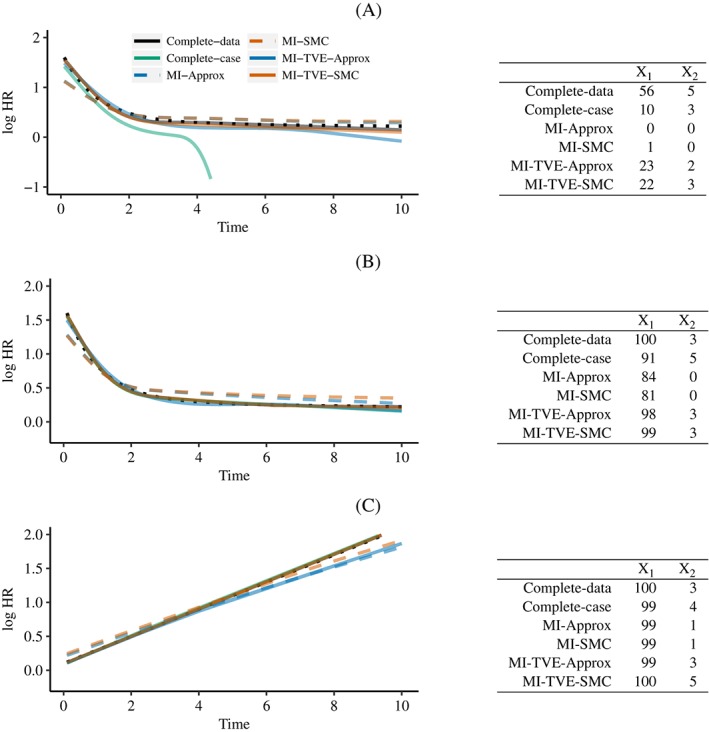
Results from additional simulations. The plots show the curvewise estimates of TVEs for covariate X_1_. The dotted black line indicates the true curve. The tables shows the percentage of simulations in which the null hypotheses of proportional hazards for X_1_ and X_2_ were rejected using joint Wald tests. A, Scenario 4, binary X_1_ and X_2_: The probability of missingness in X_1_ and X_2_ depends on D; B, Scenario 4, binary X_1_ and X_2_: 50% of individuals had the event; C, Scenario 2, continuous X_1_ and X_2_: the proportion of individuals missing X1 or X2 was reduced to 20%. MI, multiple imputation; SMC, substantive‐model‐compatible; TVE, time‐varying effect [Colour figure can be viewed at http://wileyonlinelibrary.com]

Other aspects of the simulations were as described above. Results are presented in Figure 4. For additional simulations (A) and (B), results are presented for scenario 4 with binary covariates, representing a situation in which the association between *X*_1_ and the hazard becomes weaker over time. For additional simulation (C), we focused on scenario 2 with continuous covariates, for which we found biased estimates using MI‐TVE‐Approx in the earlier simulation results.

When the missingness depends on the outcome the complete‐case analysis gives biased estimates. The results show that the proposed MI methods continue to perform well, while ignoring TVEs in the imputation still results in bias, as we would expect on the basis of our earlier results. The proposed methods also continue to perform well in a situation in which 50% of individuals have the event. When the proportion of individuals with missing data is reduced, the bias from MI‐TVE‐Approx in scenario 2 with continuous covariates is reduced but still evident when the time‐varying effect is large.

## 6 ILLUSTRATION: ROTTERDAM BREAST CANCER STUDY

We illustrate the methods using data on 2982 individuals with primary breast cancer from the Rotterdam tumour bank. The data set is freely available (we used the data set provided at https://www.imbi.uni-freiburg.de/Royston-Sauerbrei-book/index.html#datasets). This data set was chosen for two principal reasons: (i) it has been used by other authors to illustrate methods for handling TVEs^12^, ^42^; (ii) the data set being freely available, readers can use our code provided at https://github.com/ruthkeogh/MI-TVE to verify the analysis and change it for their own purposes.

Individuals were followed up from the time of breast cancer diagnosis to *disease recurrence* or *death due to breast cancer* (a composite event). Follow‐up ranged from 1 to 231 months, and 1518 individuals (51%) had the outcome of interest. Following previous applications,^12^, ^42^ we focus on the following eight covariates: age, tumour size 1 (≤ 20mm, > 20mm), tumour size 2 (≤ 50mm, > 50mm), tumour grade (grade 2 or 3 vs.  grade 1), number of positive lymph nodes (transformed as exp(−2×0.12×no.positivelymphnodes)), treatment with hormonal therapy (yes vs no), treatment with chemotherapy (yes vs. no), and progesterone receptors (pmol/l) (transformed as log(progestoronereceptors+1)).

We generated missing data at random (MAR) in five covariates (tumour grade, number of positive lymph nodes, hormonal therapy, chemotherapy, and progesterone receptors) with the probability of missingness depending on age and tumour size (probability of missingness *e* ^−9 + 0.1 × age − tumour size 2^/(1 + *e* ^−9 + 0.1 × age − tumour size 2^)). The missing data were generated conditionally independently (given age and tumour size 2) for each variable such that approximately 5% of individuals have missing data in any given variable. This resulted in 620 individuals (20% of the cohort) having missing data on at least one covariate.

A complete‐data analysis was performed before missingness was introduced and a complete‐case analysis was performed on the subset of 2362 individuals with no missing data. MI analyses were performed using the four methods considered in this paper: MI‐Approx, MI‐SMC, MI‐TVE‐Approx, and MI‐TVE‐SMC. All MI analyses used 20 imputations. All models considered included all eight covariates.

We first fitted a Cox regression model including TVEs for all variables, modelled using restricted cubic splines with 5 knots at the 5th, 25th, 50th, 75th, and 95th percentiles of the event times (0.51, 1.30, 2.54, 4.60, 9.12 years). Secondly, a test of the proportional hazards assumption was performed for each covariate using a joint Wald test (Section 4). Thirdly, we applied the MI‐MTVE algorithm described in Section 4. The same procedure (steps 2‐4 of the algorithm) was used in complete‐data and complete‐case analyses: in step 2, the complete‐data or complete‐case data set is used in place of the imputed data sets, and in step 3, standard joint Wald tests are used. A p‐value cut‐off of *α* = 0.01 was used in the TVE model selection.

**Figure 5:**
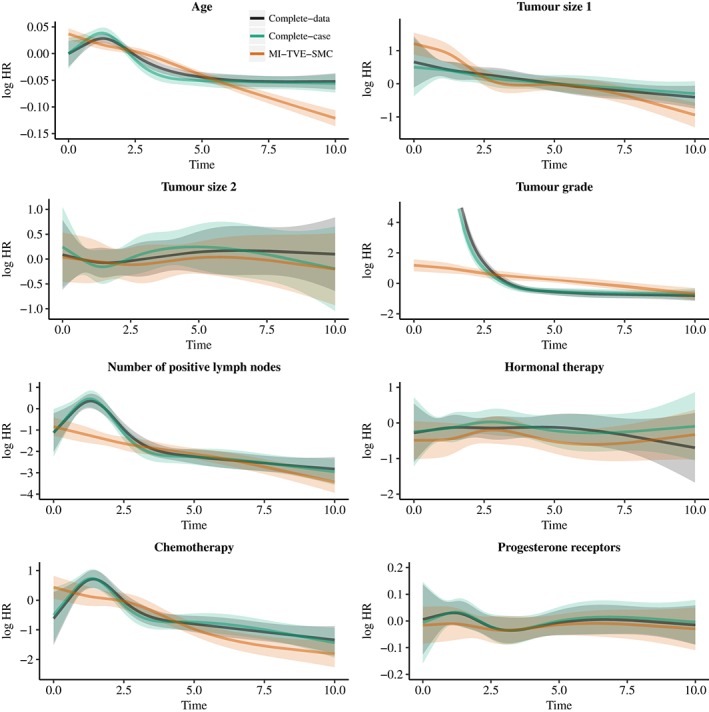
Results from the Rotterdam Breast Cancer Study. Plots showing estimated log hazard ratios as a function of time from a complete‐data analysis, complete‐case analysis, and MI‐TVE‐SMC analysis. The time‐varying effects for all covariates were modelled using a restricted cubic spline with 5 knots. Shaded errors show 95% confidence intervals. Results are shown up to time 10. MI, multiple imputation; SMC, substantive‐model‐compatible; TVE, time‐varying effect [Colour figure can be viewed at http://wileyonlinelibrary.com]

The results are shown in Figure 5, Supplementary Figure S9, and Table 2. On the basis of tests of the proportional hazard assumption, all analysis methods found evidence of TVEs for age, tumour size 1, tumour grade, number of positive lymph nodes, and chemotherapy (Table 2). There was little evidence of a TVE for tumour size 2, hormonal therapy, or progesterone receptors, except for progesterone receptors using MI‐Approx. The form of TVEs was found to be broadly similar across all methods (Figure 5, Supplementary Figure S9). The complete‐data and complete‐case analyses gave very similar results. This is not unexpected here beause the data were generated to be missing dependent only on covariates and not the outcome. Results from MI‐Approx, MI‐TVE‐Approx, and MI‐SMC‐TVE were almost identical, whereas results from MI‐SMC tended to differ from results of the other MI analyses. The confidence intervals obtained using our favoured imputation approach, MI‐TVE‐SMC (Figure 5), are not very noticeably narrower than from the complete‐case analysis in this example, though in general, as found in the simulation study, the MI analysis will be more efficient. Older age was associated with an increasingly negative association with the hazard over time. Larger tumour size was associated with an increased hazard at the start of follow‐up, which reduced over time. Those with tumour grade 2 or 3 had a much increased hazard relative to those with tumour grade 1 up to around 3 years of follow‐up, after which a higher tumour grade was associated with a slightly reduced hazard. A higher number of positive lymph nodes was associated with higher hazard (noting that the variable is transformed in the analyses), and the association became stronger with increased follow‐up. At the start of follow‐up, chemotherapy was associated with an increased hazard but in later follow‐up was associated with an increasingly reduced hazard.

**Table 2:** Results from the Rotterdam Breast Cancer Study. (i) P‐value from a joint Wald test of the null hypothesis of no TVE for that covariate, based on a model in which TVEs were modelled using restricted cubic splines with 5 knots. (ii) Indication of whether TVEs for that covariate were included ( ✓) or excluded (✗) using the MI‐MTVE algorithm, and if included, the functional form of the TVE^∗^.^∗^‘lin’ denotes a TVE of linear form f_X_(t;***β***) = β_0_ + β_1_ t. “k3”, “k4”, and “k5” denote restricted cubic spline forms for the TVE with 3, 4, and 5 knots

Covariate	Complete‐data		Complete‐case		MI‐Approx		MI‐SMC		MI‐TVE‐Approx		MI‐TVE‐SMC	
	(i)	(ii)	(i)	(ii)	(i)	(ii)	(i)	(ii)	(i)	(ii)	(i)	(ii)
Age	<0.001	✓(k5)	<0.001	✓(k5)	<0.001	✓(k5)	<0.001	✓(k5)	<0.001	✓(k5)	<0.001	✓(k5)
Tumour size 1	<0.001	✓(lin)	0.025	✓(lin)	<0.001	✓(k5)	<0.001	✓(k3)	<0.001	✓(k5)	<0.001	✓(k5)
Tumour size 2	0.941	✗	0.678	✗	0.077	✓(k3)	0.089	✗	0.850	✓(k3)	0.086	✓(k3)
Tumour grade	<0.001	✓(k5)	<0.001	✓(k5)	<0.001	✓(lin)	<0.001	✓(k5)	<0.001	✓(lin)	<0.001	✓(lin)
No. positive lymph nodes	<0.001	✓(k5)	<0.001	✓(k5)	<0.001	✓(k3)	<0.001	✓(k5)	<0.001	✓(k3)	<0.001	✓(k3)
Hormonal therapy	0.867	✗	0.919	✗	0.504	✗	0.543	✗	0.576	✗	0.553	✗
Chemotherapy	<0.001	✓(k5)	0.364	✓(k5)	<0.001	✓(k5)	0.589	✓(k5)	<0.001	✓(k5)	<0.001	✓(k5)
Progesterone receptors	0.478	✗	0.600	✗	0.158	✗	0.117	✓(k5)	0.010	✗	0.119	✗

Abbreviations: MI, multiple imputation; SMC, substantive‐model‐compatible; TVE, time‐varying effect.

The MI‐MTVE algorithm selected TVEs for the same variables using all methods (Table 2), with the exception of tumour size 2, which was selected to have a TVE using MI‐Approx, MI‐TVE‐Approx and MI‐TVE‐SMC but not the other methods and progesterone receptors, which was selected to have a TVE by MI‐SMC. The functional forms chosen by the algorithm differed somewhat across methods.

In summary, similar conclusions would be reached from all analyses in this example. Further investigations looking at different missing data mechanisms and using different example data sets would be of interest. Sauerbrei et al^12^, ^43^ applied the MFPT variable selection procedure on the basis of fractional polynomial models to this data set (using the complete data), identifying TVEs for tumour size 1 and progesterone receptors. Further work on how best to perform variable selection incorporating TVEs is needed even without the complication of missing data.

## 7 DISCUSSION

In this article, we have introduced two multiple imputation methods allowing for TVEs to be included in Cox regression models. In the absence of TVEs, the methods of White and Royston^5^ (MI‐Approx) and Bartlett et al^6^ (MI‐SMC) can be used. MI‐Approx is conceptually simpler, more convenient to code, and faster to run, whereas MI‐SMC method has better statistical properties. Our two proposals are extensions of these methods. The methods were described for a general functional form for TVEs. Researchers use different approaches to modelling TVEs. The correct functional form for a TVE is typically not known in advance, and thus, it is desirable to allow a flexible form. We therefore focused on a situation in which TVEs are modelled using restricted cubic splines. In some studies, it may be desirable for the TVE to be a simple step function, and we provided details on this in the Supplementary Materials (Section S3). In simulation studies, we used imputation models assuming a five‐knot restricted cubic spline for the TVE. For binary covariates with missing data, both of our proposed methods performed well. However, the performance of the approximate method (MI‐TVE‐Approx) was slightly disappointing for continuous covariates when the effect size is large, though it still outperformed the complete‐case analysis in all but scenario 2, and the observed bias was found to be smaller when the proportion with missing data is lower. The SMC method (MI‐TVE‐SMC) performed well across all scenarios, minimising bias, retaining approximately correct type I error rates in tests for nonproportional hazards when there is no TVE, and maximising power to detect TVEs when they exist compared with other MI methods. Our results showed that ignoring TVEs in the imputation model will result in incorrect type I errors (that is, not equal to the nominal level *α*) in the test for nonproportional hazards when the null hypothesis of proportional hazards is true and in a large loss of power to detect a TVE when one exists.

In practice, TVEs will often appear in the context of model building, including tests of the proportional hazards assumption. We therefore proposed the MI‐MTVE model selection algorithm, an adaptation the MFPT algorithm of Sauerbrei et al,^12^ for such settings. There remains work to be done to assess the performance of the MI‐MTVE model selection algorithm and to compare it with possible alternatives. Our procedures for testing the proportional hazard assumption and the MI‐MTVE algorithm draw on earlier work by Wood et al^33^ on variable selection methods using multiply imputed data. There is a sizeable literature on model selection incorporating estimation of TVEs without including treatment of missing data. Berger et al^44^ proposed the use of fractional polynomials^45^ to select parsimonious forms for TVEs in Cox regression. Sauerbrei et al^12^ proposed a model selection algorithm for use in Cox regression in which both the functional form for continuous covariates and the functional form for TVEs of covariates are modelled using fractional polynomials (the MFPT algorithm). See also chapter 11 in Royston and Sauerbrei.^42^ Abrahamowicz et al^10^ and Wynant and Abrahamowicz^11^ also described methods for joint estimation of time‐varying and nonlinear effects based on splines. Areas for further work include the extension of the methods proposed in this paper to a setting in which TVEs are modelled using fractional polynomials and to allow selection of functional forms for continuous variables and covariate interactions. This will build on the work Morris et al^34^ on how to incorporate MI into a fractional polynomial model building procedure for explanatory variables. The MI‐TVE‐SMC approach is particularly suitable for extensions involving transformed covariates. Finally, further work is needed to investigate the validity of inferences following data‐dependent model selection processes in the missing data context.

We have provided R code (https://github.com/ruthkeogh/MI-TVE), which can be used to implement the proposed imputation methods and perform analyses of the example data set used in Section 6. Moreover, MI‐TVE‐Approx is straightforward to apply in standard software, and although we provide example R code, this method can also be easily applied in Stata (mi impute) or SAS (PROC MI), for example. In addition, MI‐SMC (not incorporating TVEs) can be applied using the smcfcs packages in R and Stata.^46^ We have also provided an adaptation of this code for implementing MI‐TVE‐SMC in R. We applied our proposed methods to the analysis of the Rotterdam breast cancer study data, followed by the MI‐MTVE model selection algorithm. The methods led to similar conclusions in this case. The data set is freely available, and we provide code at the above webpage, which enables the user to verify the analysis and change it for their own purposes.

There are of course limitations to this work. In particular, in some settings, complete‐case analysis will be unbiased and MI biased. It follows that our methods are only applicable to settings in which MI is judged to be the best approach. When using our proposed methods, other forms of misspecification of the imputation model could result in bias, and this should be borne in mind, as in any MI analysis, especially for partially missing variables that are continuous, for which the normality assumption may not hold. Further work is also needed to investigate the performance of different approaches to model selection in this context. The general results given for MI‐TVE‐Approx and MI‐TVE‐SMC assumed that any censoring occurs independently of covariates with missing data. In MI‐TVE‐Approx, censoring depending on covariates with missing data can be accommodated by adding a further term, ${\hat{H}}_{C}(T\;)$, into the imputation model, which denotes the Nelson‐Aalen estimate of the cumulative hazard for the censoring.^17^ In addition, MI‐SMC has been extended to allow competing risks,^47^ and this can be used to handle dependence of right‐censoring on variables with missing data by modelling the censoring as a competing event. Moreover, MI‐TVE‐SMC can be extended in the same way. In both cases, it is assumed that the association between covariates and the hazard for censoring is not time‐varying. Event times are also commonly subject to left truncation. Using MI‐TVE‐Approx, it can be shown that left truncation can be accommodated by replacing $\hat{H}(T\;)$ by $\hat{H}(T)-\hat{H}(T_{L})$ (and ${\hat{H}}^{\left(1\right)}(T)$ by ${\hat{H}}^{\left(1\right)}(T)-{\hat{H}}^{\left(1\right)}(T_{L})$) in the imputation model and ${\hat{H}}_{C}(T)$ by ${\hat{H}}_{C}(T)-{\hat{H}}_{C}(T_{L})$ if the censoring is suspected to depend on partially missing covariates. MI‐TVE‐SMC can also be extended to accommodate left truncation; however, further work is needed on this topic, including to implement the methods in the software.

We have focused on estimation of TVEs by modelling these in the Cox regression model. There exist other methods for estimating and testing for TVEs in Cox regression. Schoenfeld residual plots can be used to visually assess the proportional hazards assumption,^48^ and smoothed residuals can be used to estimate TVEs.^49^, ^50^ Scheike and Martinussen^51^ outlined tests for proportional hazards based on an iterative procedure to estimate cumulative regression coefficients, which does not require specification of the functional form for TVEs. Ng'andu^52^ summarized and compared several tests for the proportional hazards assumption. Other methods for estimating TVEs include those based on a kernel‐weighted local partial likelihood^53^ and penalized partial likelihoods.^54^, ^55^, ^56^ Gerds and Schumacher^57^ proposed a measure for use in choosing between different models for the TVE to discover which is closest to the true shape.

In summary, for settings in which MI is judged to be appropriate and TVEs are a feature of the analysis, the approaches we have described should be used. Where computational time is not too large, the MI‐TVE‐SMC approach is recommended, though MI‐TVE‐Approx should also perform well if all covariates with missingness are binary or if effect sizes are small. Ignoring TVEs in the imputation may result in biased estimates and misleading conclusions.

## Supporting information

supplementary_materials_revision2.pdf
