# Supplementary material for: Multiple imputation in Cox regression when there are time‐varying effects of covariates
Source: Stat Med. 2018 Jul 16;37(25):3661–78. doi: 10.1002/sim.7842 (PMC6220767; doi:10.1002/sim.7842)

Multiple imputation in Cox regression when there are time-varying effects of exposures: Supplementary materials

Ruth H. Keogh[a], Tim P. Morris[b]

[a] Department of Medical Statistics, London School of Hygiene and Tropical Medicine, Keppel Street, London WC1E 7HT, UK.

[b] London Hub for Trials Methodology Research, MRC Clinical Trials Unit at UCL, Aviation House, 125 Kingsway, London WC2B 6NH, UK.

Multiple imputation in Cox regression when there are time-varying effects of exposures: Supplementary materials

S1. Derivation of imputation models in MI-TVE-Approx

The focus is on a single explanatory variable X_1 with missing data and a fully observed covariate, X_2. The hazard model of interest is $h(t|X_1, X_2) = h_0(t)\exp\{f_{X_1}(t; \boldsymbol{\beta}_{X1})X + f_{X_2}(t; \boldsymbol{\beta}_{X2})X_2\}$. In the context of Cox regression, MI relies of obtaining draws of missing values of X_1 from its distribution given T, D, X_2. The probability density function for the conditional distribution of X_1, which we denote by $p(X_1|T, D, X_2)$, can be expressed as

$$p(X_1|T, D, X_2) = p(T, D|X_1, X_2)p(X_1|X_2)/p(T, D|X_2). \tag{S1}$$

The first term can be written as

$$p(T, D|X_1, X_2) = h(T|X_1, X_2)^D S(T|X_1, X_2) h_C(T|X_1, X_2)^{1-D} S_C(T|X_1, X_2) \tag{S2}$$

where $S(.)$ is the survivor function for the event of interest, $h_C(t|X_1, X_2)$ is the hazard for censoring, and $S_C(.)$ is the survivor function corresponding to the censoring process. We assume for now that any censoring occurs independently of X_1 and so the third and fourth terms of (S2) can be ignored in the workings which follow, since they do not involve X_1, and so can be subsumed into a constant of proportionality. Details on handling censoring which depends on X_1 are given in the Discussion section of the paper. Under the hazard model of interest we have

$$\begin{aligned}\log p(T, D|X_1, X_2) = D\log h_0(T) + D\{f_{X1}(T, \beta_{X1})X_1 + f_{X2}(T, \beta_{X2})X_2\} \\ - \int_0^T h_0(u) e^{f_{X1}(u,\beta_{X1})X_1 + f_{X2}(u,\beta_{X2})X_2} \mathrm{d}u.\end{aligned} \tag{S3}$$

It follows that

$$\begin{aligned}\log p(X_1|T,D,X_2) &= \log p(X_1|X_2) + Df_{X1}(T,\beta_{X1})X_1 \\ &- \int_0^T h_0(u)e^{f_{X1}(u,\beta_{X1})X_1+f_{X2}(u,\beta_{X2})X_2}\mathrm{d}u + q(T,D,X_2)\end{aligned} \tag{S4}$$

where $q(T,D,X_2)$ represents terms not involving X_1.

In the situation with time varying effects the expression $\log p(X_1|T,D,X_2)$ is complicated by the presence of $e^{f_{X1}(u,\beta_{X1})X_1+f_{X2}(u,\beta_{X2})X_2}$ in the integral. For some forms for the TVE functions a closed form solution to the integral would be possible, however a more general result is desirable. A linear approximation to $e^{f_{X1}(u,\beta_{X1})X_1+f_{X2}(u,\beta_{X2})X_2}$ is therefore used. The linear approximation is:

$$\begin{aligned}e^{f_{X1}(u,\beta_{X1})X_1+f_{X2}(u,\beta_{X2})X_2} \approx\, & e^{f_{X1}(\bar{u},\beta_{X1})X_1+f_{X2}(\bar{u},\beta_{X2})X_2} + (u-\bar{u})\{f'_{X1}(\bar{u},\beta_{X1})X_1 \\ & +f'_{X2}(u,\beta_{X2})X_2\}e^{f_{X1}(\bar{u},\beta_{X1})X_1+f_{X2}(\bar{u},\beta_{X2})X_2}\end{aligned} \tag{S5}$$

where $\bar{u}$ denotes the mean of the observed event times. The approximation is expected to perform well when the TVEs are not too large, i.e. when the log hazard ratios at any given time are not too large. Higher order approximations could be considered, and in Section S3 we consider a stepwise approximation. In the results given below, we let
$A(X_1,X_2) = f_{X1}(\bar{u},\beta_{X1})X_1 + f_{X2}(\bar{u},\beta_{X2})X_2$,
$B(X_1,X_2) = f'_{X1}(\bar{u},\beta_{X1})X_1 + f'_{X2}(\bar{u},\beta_{X2})X_2$.

To proceed, it is necessary to make some assumptions about $p(X_1|X_2)$. Next, we consider the situations of binary X_1 and Normally distributed X_1.

S1.1 Binary X_1

We assume a logistic model for X_1 given X_2:

$$\text{logit } p(X_1 = 1|X_2) = \zeta_0 + \zeta_1 X_2. \tag{S6}$$

First, suppose that X_2 is also binary. Then, using the approximation in (S5), it can be shown that

$$\begin{aligned} \text{logit } p(X_1 = 1|T, D, X_2) &\approx \zeta_0 + \zeta_1 X_2 + D \times f_{X1}(T; \beta_{X1}) \\ +H_0(T)\{A(0, X_2) - A(1, X_2) + \bar{u}A(1, X_2)B(1, X_2) &- \bar{u}A(0, X_2)B(0, X_2)\} \\ +H_1(T)\{A(0, X_2)B(0, X_2) &- A(1, X_2)B(1, X_2)\} \end{aligned} \tag{S7}$$

where $H_0(T)$ denotes the cumulative baseline hazard and $H_1(T) = \int_0^T uh_0(u)du$.

If X_2 is continuous, we use a bivariate linear approximation to $e^{f_{X1}(u,\beta_{X1})X_1 + f_{X2}(u,\beta_{X2})X_2}$, about $\bar{u}$ and $\bar{X}_2$ (the sample mean of X_2). It can be shown that in this case

$$\begin{aligned} \text{logit } p(X_1 = 1|t, d, X_2) &\approx \zeta_0 + \zeta_1 X_2 + d \times f_{X1}(t; \beta_{X1}) \\ +H_0(t)\{-A(1, \bar{X}_2) &- A(1, \bar{X}_2)f_{X2}(\bar{u}; \beta_{X2})(X_2 - \bar{X}_2) \\ +A(0, \bar{X}_2) &+ A(0, \bar{X}_2)f_{X2}(\bar{u}; \beta_{X2})(X_2 - \bar{X}_2) \\ +\bar{u}A(1, \bar{X}_2)B(1, \bar{X}_2) &- \bar{u}A(0, \bar{X}_2)B(0, \bar{X}_2)\} \\ +H_1(t)\{A(0, \bar{X}_2)B(0, \bar{X}_2) &- A(1, \bar{X}_2)B(1, \bar{X}_2)\} \end{aligned} \tag{S8}$$

It follows from the expressions in (S7) and (S8) that an approximate imputation model for X_1 is a logistic regression for X_1 with main effects of X_2, $H_0(T)$, $H_1(T)$, the interaction between D and $f_{X1}(T)$, and interactions of X_2 with $H_0(T)$ and $H_1(T)$. If the TVE function is $f_{X1}(t; \beta_{X1}) = \beta_{X01} + \beta_{X11}t$, for example, the imputation model should include D and the interaction between D and T. In the case of a restricted cubic spline with $L = 5$ knots, the imputation model should include D and the interaction between D and T and interactions of D with $\left\{(T - u_i)_+^3 - \left(\frac{(T-u_{L-1})_+^3(u_L-u_i)}{(u_L-u_{L-1})}\right) + \left(\frac{(T-u_L)_+^3(u_{L-1}-u_i)}{(u_L-u_{L-1})}\right)\right\}$ for $i = 1, 2, 3$.

In the situation without TVEs, the above results reduce to those of White and Royston (2009). The imputation models involve the baseline cumulative hazard $H_0(T)$ and the additional integral term $H_1(T)$. When there are no TVEs, the imputation model includes only $H_0(T)$ and White and Royston (2009) White and Royston (2009)

suggested replacing this with the Nelson-Aalen estimate of the cumulative hazard, $\widehat{H}(T) = \sum_{t \leq T} \frac{d(t)}{n(t)}$, where $d(t)$ is the number of events at time t and $n(t)$ is the number of individuals at risk at time t. This has been found to perform at least as well as a more complex method using Breslow's estimate for $H_0(T)$ in simulation studies. Following similar reasoning, we propose using the Nelson-Aalen-type estimator $\widehat{H}^{(1)}(T) = \sum_{t \leq T} \frac{td(t)}{n(t)}$ in place of $H_1(T)$.

S1.2 Continuous X

To derive an imputation model for a continuous X_1 we assume that, conditionally on X_2, X_1 is normally distributed with mean $\zeta_0 + \zeta_1 X_2$ and variance σ^2. The derivations, which are not shown in detail here, use a quadratic (i.e. second order) trivariate approximation for $e^{f_{X1}(u,\beta_{X1})X_1 + f_{X2}(u,\beta_{X2})X_2}$ about $\bar{u}$, $\bar{X}_1$ and $\bar{X}_2$. It can be shown that an approximate imputation model for X_1 is a a linear regression of X_1 with main effects of X_2, $H_0(T)$, $H_1(T)$, the interaction between D and $f_{X1}(T)$, and interactions of X_2 with $H_0(T)$ and $H_1(T)$. That is, the imputation model contains the same terms as for binary X_1 described above. AS above, we propose replacing $H_0(T)$ and $H_1(T)$ with the estimates $\widehat{H}(T)$ and $\widehat{H}^{(1)}$ respectively.

S2. Extensions to MI-TVE-Approx and MI-TVE-SMC: handling missing data in more than one covariate using full conditional specification (FCS)

In this section we describe extensions to MI-TVE-Approx and MI-TVE-SMC for the situation with more than one covariate with missing data. Let $X = (X_1, X_2, \ldots, X_p)'$ denotes the vector of partially observed variables. The model of interest is assumed to be of the form

$$h(t|X) = h_0(t) \exp\left\{ \sum_k f_{X_k}(t; \boldsymbol{\beta}_{Xk}) X_k \right\}$$

Additional fully observed covariates can be incorporated in a straightforward manner. For MI-TVE-approx, the FCS algorithm to generate a single imputed dataset is as follows.

1. Replace the missing values in X by arbitrary starting values, to create a complete data set. In practice, one could replace missing values of X_k ($k = 1, \ldots, p$) by the mean of X_k among those individuals in whom X_k is observed. Set $k = 1$.

2. If X_k is a continuous variable, fit the imputation model

$$X_k = \alpha_0+\alpha_1' X_{-k}+\alpha_2^T D f_{Xk}(T)+\alpha_3\widehat{H}(T)+\alpha_4\widehat{H}^{(1)}(T)+\alpha_5' X_{-k}\widehat{H}(T)+\alpha_6' X_{-k}\widehat{H}^{(1)}(T)+\epsilon,$$

   with residual error variance σ_ϵ^2, to the subset of individuals for whom X_k is observed, using the current values of X_{-k}. If X_k is a binary variable, the imputation model is the logistic regression

$$\begin{aligned}\text{logit}\ \Pr(X_k = 1|T, D, X_{-k}) = \alpha_0 + \alpha_1' X_{-k} + \alpha_2^T D f_{Xk}(T) + \alpha_3\widehat{H}(T)\\ +\alpha_4\widehat{H}^{(1)}(T) + \alpha_5' X_{-k}\widehat{H}(T) + \alpha_6' X_{-k}\widehat{H}^{(1)}(T).\end{aligned}$$

   Take a random draw $(\alpha_0^*, \alpha_1^*, \alpha_2^*, \alpha_3^*, \alpha_4^*, \alpha_5^*, \alpha_6^*, \sigma_\epsilon^{2*})$ (if X_k is continuous) or $(\alpha_0^*, \alpha_1^*, \alpha_2^*, \alpha_3^*, \alpha_4^*, \alpha_5^*, \alpha_6^*)$ (if X_k is binary) from the approximate posterior distribution of the parameters in this model.

3. If X_k is continuous, then for each individual with missing X_k in the original data set, replace the current value of X_k with a sample from a normal distribution with mean
$\alpha_0^* + \alpha_1'^* X_{-k} + \alpha_2'^* D f_{X1}(T) + \alpha_3^*\widehat{H}(T) + \alpha_4^*\widehat{H}^{(1)}(T) + \alpha_5'^* X_{-k}\widehat{H}(T) + \alpha_6'^* X_{-k}\widehat{H}^{(1)}(T)$
and variance σ_ϵ^{2*}. If X_k is binary, sample instead from a Bernoulli distribution with the same mean.

4. If $k < p$, set $k = k + 1$ and return to step 2.

Repeat steps 2–4 until the sampled values of X converge in distribution. At this point, use these sampled values as the imputed values for the single imputed dataset. Repeat the whole process M times to generate M imputed datasets.

For MI-TVE-SMC with p partially observed variables, the algorithm to generate one imputed data set is as follows.

1. Replace the missing missing values in X with arbitrary starting values, to create a complete dataset. Set $k = 1$.

2. Fit the Cox regression model of interest, including the TVEs, to the current complete data set to obtain estimates $\hat{\boldsymbol{\beta}}_{Xk}$ $(k = 1, \ldots, p)$ and their estimated variance $\hat{\Sigma}$. Draw values $\boldsymbol{\beta}^*_{Xk}$ $(k = 1, \ldots, p)$ from a joint normal distribution with mean $(\hat{\boldsymbol{\beta}}_{X1}, \ldots, \hat{\boldsymbol{\beta}}_{Xp})$ and variance $\hat{\Sigma}$.

3. Calculate Breslow's estimate, denoted $H^*_0(t)$, of the baseline cumulative hazard $H_0(t)$ using the parameter values $\boldsymbol{\beta}^*_{Xk}$ $(k = 1, \ldots, p)$ and the current imputations of X.

4. Fit a regression model (e.g. linear or logistic, as appropriate) of X_k on X_{-k} to the current complete data set. Draw a value γ^*_{Xk} from the approximate joint posterior distribution of the parameters γ_{Xk} in this model.

5. For each individual for whom X_k is missing, (a) draw a value X^*_k from the distribution $p(X_k|X_{-k}; \gamma^*_{Xk})$ and let X^* denote X with X_k replaced by its proposed value X^*_k, (b) draw a value U from a uniform distribution on $[0, 1]$, and (c) accept the proposal X^*_k if

$$\begin{cases} U \leq \exp\left[-\sum_{j:t_j \leq T} \Delta H_0^{(m)}(t_j) \exp\left\{\sum_k f_{Xk}\left(t_j; \boldsymbol{\beta}_{Xk}^{(m)}\right) X^*_k\right\}\right] & \text{if } D = 0 \\ U \leq \Delta H_0^{(m)}(T) \exp\left\{1 + f_{Xk}\left(T; \boldsymbol{\beta}_{Xk}^{(m)}\right) X^*_k - \sum_{j:t_j \leq T} \Delta H_0^{(m)}(t_j) e^{f_{Xk}\left(t_j; \boldsymbol{\beta}_{Xk}^{(m)}\right) X^*_k}\right\} & \text{if } D = 1 \end{cases}$$

   If X^*_k is not accepted, then discard it and repeat (a), (b) and (c).

6. If $k < p$, let $k = k + 1$ and return to step 2.

Repeat steps 2–6 until the sampled values of X converge in distribution. At this point, use these sampled values as the imputed values for the single imputed dataset. Repeat the whole process M times to generate M imputed datasets.

S3. Using a step-function form for the time-varying effect

A simple approach to investigating the TVE of a covariate is to assume a step function form for $f_X(t;\boldsymbol{\beta})$, such that the hazard ratio is assumed constant within a series of time periods (see for example Gore, Pocock, and Kerr (1984)). In the case, focusing on our situation with a partially observed covariate X_1 and a fully observed covariate X_2, the hazard function is

$$h(t|X_1,X_2) = h_0(t)\exp\left\{\sum_{j=1}^{K}\beta_{X1j}I_jX_1 + \sum_{j=1}^{K}\beta_{X2j}I_jX_2\right\} \tag{S9}$$

where there are K time periods $(0,s_1],(s_1,s_2],\ldots,(s_{K-1},s_K]$ and $I_k = I(s_{k-1} < t \leq s_k)$ is an indicator taking value 1 if t lies in the interval from s_{k-1} to s_k $(k=1,\ldots,K)$ and 0 otherwise. A step function is unlikely to represent the true underlying time-varying effect and more realistic models are based on splines or other flexible functional forms, which are the main focus of the paper. However, because a step-function is sometimes used, we present some brief details here. By following similar workings as shown in Section S1, it can be shown that a suitable imputation model for X_1 is a logistic regression (for binary X_1) or linear regression (for continuous X_1) on X_2, DI_k $(k=1,\ldots,K)$, H_k^* $(k=1,\ldots,K_T)$, H_T^*, $X_2H_k^*$ $(k=1,\ldots,K_T)$ and $X_2H_T^*$, where $H_k^* = \int_{s_{k-1}}^{s_k} h_0(u)\mathrm{d}u$, $H_T^* = \int_{s_T}^{T} h_0(u)\mathrm{d}u$, K_T is the number of complete time periods which have passed prior to T, and s_T is the upper limit of the last complete time period prior to T. We propose replacing H_k^* and H_T^* by their estimates $\widehat{H}_k^* = \sum_{s_{k-1}<t\leq s_k}\frac{d(t)}{n(t)}$ and $\widehat{H}_T^* = \sum_{s_T<t\leq T}\frac{d(t)}{n(t)}$. A feature of the imputation model for TVEs based on a step function is that we do not require a linear (or other) approximation to evaluate the integral in S4.

S4. Simulation study: Details on missing data generation

In the main simulation, non-monotone missing data were generated in X_1 and X_2 according to a MAR mechanism in which the probability of missingness in X_1 depends on observed values of X_2, and vice versa. To achieve this, the cohort was divided

randomly into three groups of approximately equal size. In group 1, X_2 is fully observed and X_1 was set to be missing with probability $e^{0.4+0.5X_2}/(1+e^{0.4+0.5X_2})$. In group 2, X_1 is fully observed and X_2 was set to be missing with probability $e^{0.4+0.5X_1}/(1+e^{0.4+0.5X_1})$. In group 3, X_1 and X_2 were *both* missing completely at random with probability 0.3.

In additional simulations (Section 5.5), the probability of missingness in X_1 and X_2 additionally depends on the event indicator D. The procedure described above was modified such that in group 1 the probability of missingness in X_1 was $e^{-0.4+0.5X_2+0.5D+0.5X_2D}/(1+e^{-0.4+0.5X_2+0.5D+0.5X_2D})$, in group 2 probability of missingness in X_2 was $e^{-0.4+0.5X_1+0.5D+0.5X_1D}/(1+e^{-0.4+0.5X_1+0.5D+0.5X_1D})$, and in group 3 X_1 and X_2 were *both* missing with probability $e^{-0.4+0.5D}/(1+e^{-0.4+0.5D})$.

The values used in these missing data generation procedures were selected so that X_1 is missing for approximately 30% of individuals and X_2 is missing for approximately 30% of individuals, resulting in approximately 50% of individuals missing at least one of the measurements.

In an additional simulation (Section 5.5) a lower proportion with missing data was considered. For this, in group 1, X_2 is fully observed and X_1 was set to be missing with probability $e^{-1.2+0.5X_2}/(1+e^{-1.2+0.5X_2})$. In group 2, X_1 is fully observed and X_2 was set to be missing with probability $e^{-1.2+0.5X_1}/(1+e^{-1.2+0.5X_1})$. In group 3, X_1 and X_2 were *both* missing completely at random with probability 0.1.

S5. Simulation study: Justification of number of simulated data sets

The performance measures described in Section 5.3 were used to determine the number of repetitions under each scenario. We are primarily interested in bias, and assume that the variance of bias at any given t is 0.1. Then the Monte Carlo standard error for the bias is

$$\text{MCSE} = \sqrt{\frac{\text{Var}}{\text{reps}}}. \tag{S10}$$

Aiming for MCSE of 0.015 on estimated bias, we require 445 repetitions, and rounded up to 500.

Secondary interest is in rejection fractions and coverage, for which the summary of

a simulation run is binary. Here, for rejection fraction π the MCSE is

$$\text{MCSE} = \sqrt{\frac{\pi(1-\pi)}{\text{reps}}}. \tag{S11}$$

The MCSE is maximised at $\pi = 0.5$, for which 500 repetitions returns MCSE of 2.2% , which we find acceptable. If tests have approximately the correct size, then at $\pi = 0.05$, MCSE = 1%

References

Gore, S., Pocock, S., & Kerr, G. (1984). Regression models and non-proportional hazards in the analysis of breast cancer survival. *Journal of the Royal Statistical Society (Series C)*, *33*, 176–195.

White, I. R., & Royston, P. (2009). Imputing missing covariate values for the Cox model. *Statistics in Medicine*, *28*, 1982-1998.

Table S1
Coverage of the estimated TVE curve at three time points (1, 5, 9) for covariates X_1 and X_2 in the setting with binary covariates X_1 and X_2.

	Covariate X_1			Covariate X_2		
	1	5	9	1	5	9
**Scenario 1**						
Complete-data	96	100	100	95	100	100
Complete-case	98	100	100	95	100	100
MI-Approx	99	100	100	98	100	100
MI-SMC	99	100	100	98	100	100
MI-TVE-Approx	96	100	100	96	100	100
MI-TVE-SMC	96	100	100	95	100	100
**Scenario 2**						
Complete-data	95	100	100	96	100	100
Complete-case	98	100	100	95	100	100
MI-Approx	95	100	100	98	100	100
MI-SMC	93	100	100	98	100	100
MI-TVE-Approx	96	100	100	95	100	100
MI-TVE-SMC	94	100	100	94	100	100
**Scenario 3**						
Complete-data	94	100	100	95	100	100
Complete-case	95	100	100	96	100	100
MI-Approx	98	100	100	98	100	100
MI-SMC	98	100	100	98	100	100
MI-TVE-Approx	95	100	100	96	100	100
MI-TVE-SMC	95	100	100	95	100	100
**Scenario 4**						
Complete-data	96	100	100	97	100	100
Complete-case	96	100	100	96	100	100
MI-Approx	99	100	100	99	100	100
MI-SMC	99	100	100	99	100	100
MI-TVE-Approx	98	100	100	98	100	100
MI-TVE-SMC	97	100	100	97	100	100
**Scenario 5**						
Complete-data	95	100	100	95	100	100
Complete-case	96	100	100	95	100	100
MI-Approx	99	100	100	98	100	100
MI-SMC	99	100	100	98	100	100
MI-TVE-Approx	95	100	100	95	100	100
MI-TVE-SMC	95	100	100	96	100	100

Table S2
Coverage of the estimated TVE curve at three time points (1, 5, 9) for covariates X_1 and X_2 in the setting with continuous covariates X_1 and X_2.

	Covariate X_1			Covariate X_2		
	1	5	9	1	5	9
**Scenario 1**						
Complete-data	96	100	100	96	100	100
Complete-case	95	100	100	97	100	100
MI-Approx	99	100	100	99	100	100
MI-SMC	99	100	100	99	100	100
MI-TVE-Approx	96	100	100	96	100	100
MI-TVE-SMC	97	100	100	96	100	100
**Scenario 2**						
Complete-data	94	100	100	94	100	100
Complete-case	96	100	100	96	100	100
MI-Approx	92	100	100	97	100	100
MI-SMC	86	100	100	97	100	100
MI-TVE-Approx	95	100	100	95	100	100
MI-TVE-SMC	95	100	100	95	100	100
**Scenario 3**						
Complete-data	94	100	100	96	100	100
Complete-case	95	100	100	95	100	100
MI-Approx	99	100	100	98	100	100
MI-SMC	98	100	100	99	100	100
MI-TVE-Approx	93	100	100	96	100	100
MI-TVE-SMC	95	100	100	96	100	100
**Scenario 4**						
Complete-data	100	100	100	100	100	100
Complete-case	100	100	100	100	100	100
MI-Approx	100	100	100	100	100	100
MI-SMC	100	100	100	100	100	100
MI-TVE-Approx	100	100	100	100	100	100
MI-TVE-SMC	100	100	100	100	100	100
**Scenario 5**						
Complete-data	98	100	100	99	100	100
Complete-case	99	100	100	98	100	100
MI-Approx	100	100	100	100	100	100
MI-SMC	100	100	100	100	100	100
MI-TVE-Approx	99	100	100	99	100	100
MI-TVE-SMC	97	100	100	98	100	100

Figure S1. Bias in the estimated TVE curve at three time points for covariate X_1 in the setting with binary covariates X_1 (black) and X_2 (grey). The point indicates the bias and the bar indicates the 95% confidence interval.

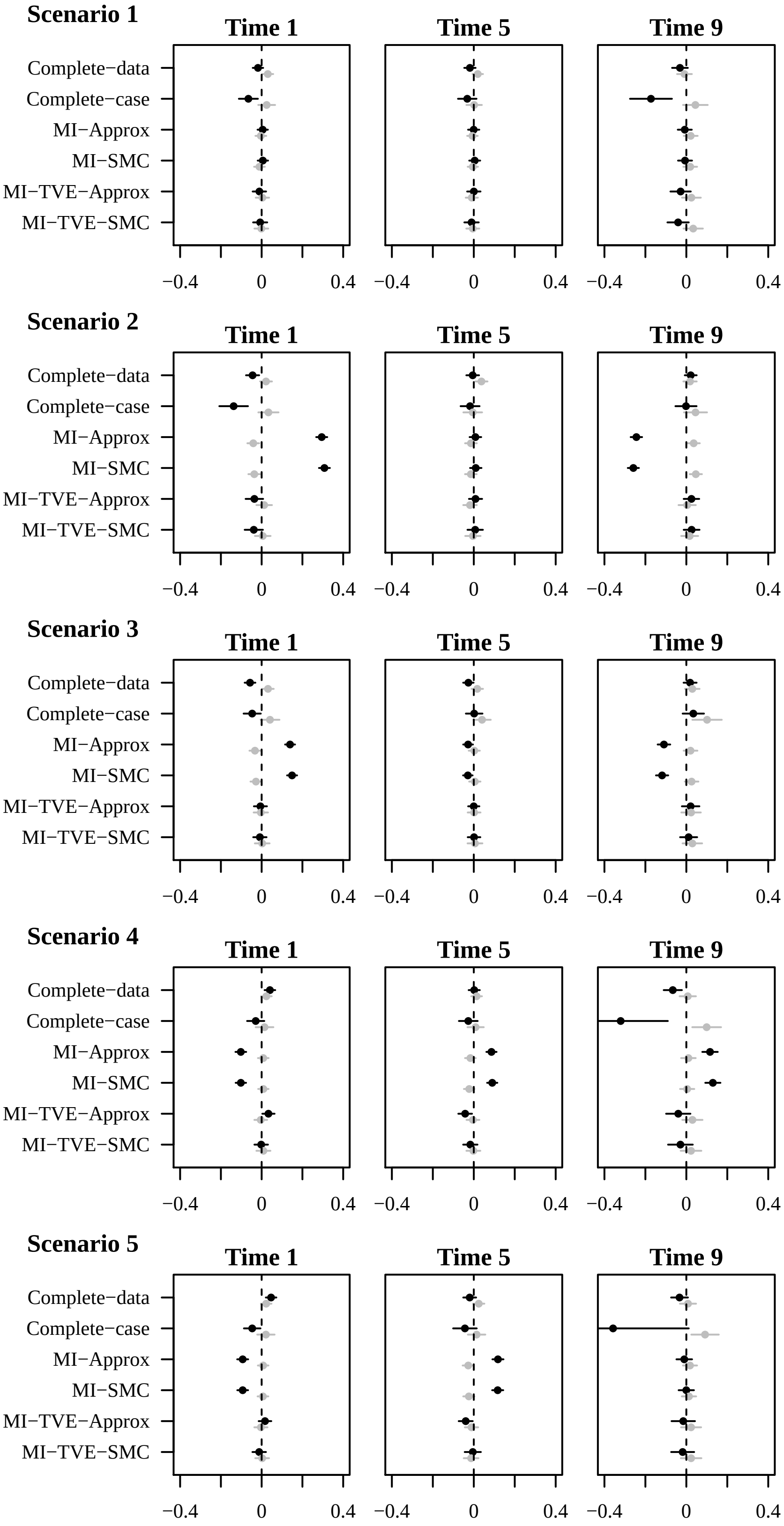

Figure S2. Bias in the estimated TVE curve at three time points for covariate X_1 in the setting with continuous covariates X_1 (black) and X_2 (grey). The point indicates the bias and the bar indicates the 95% confidence interval.

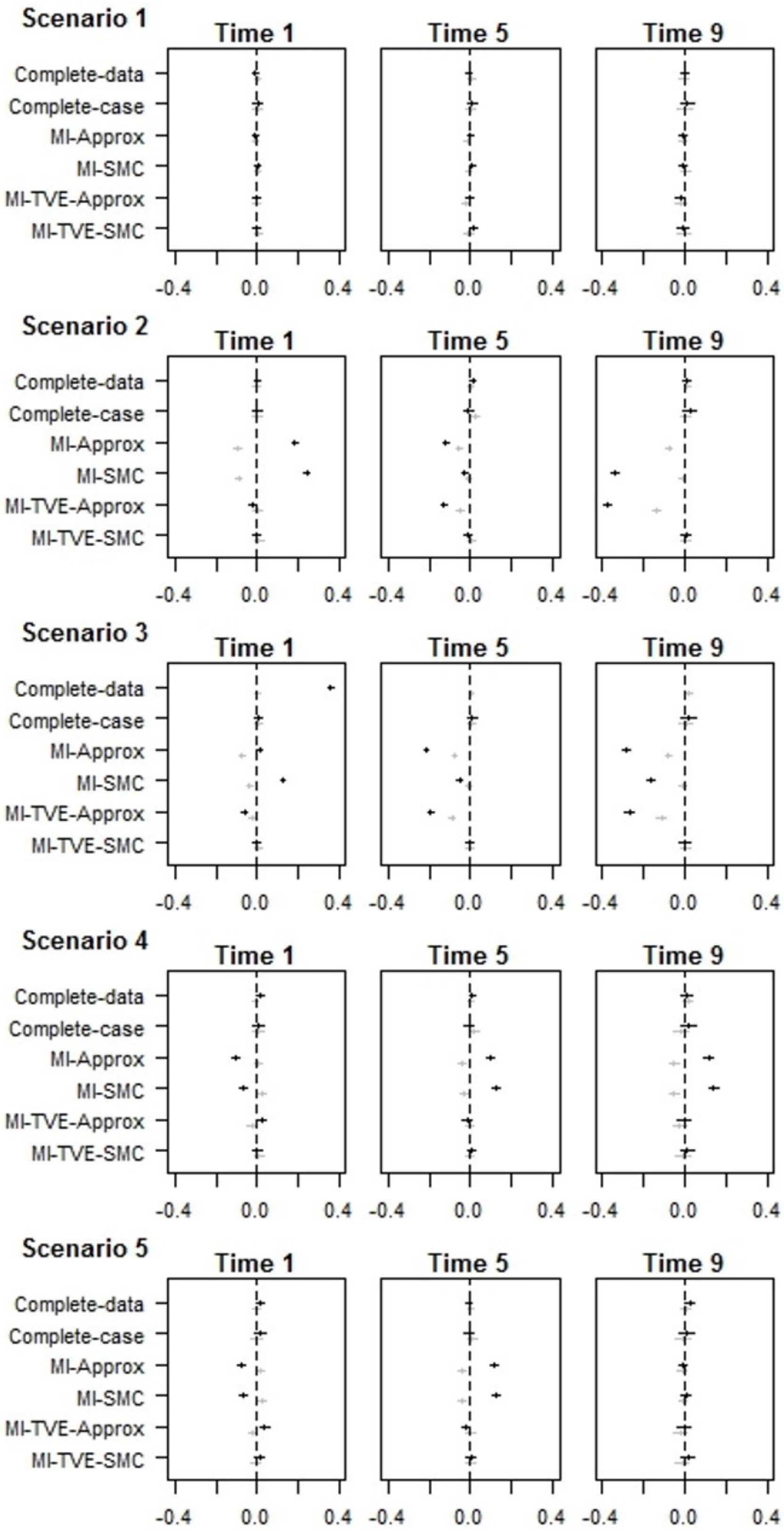

Figure S3. Curve-wise estimates of TVEs for covariate X_2 in the setting with binary covariates X_1 and X_2. The dotted black line indicates the true curve. The curves are all approximately flat because X_2 always has a non-TVE.

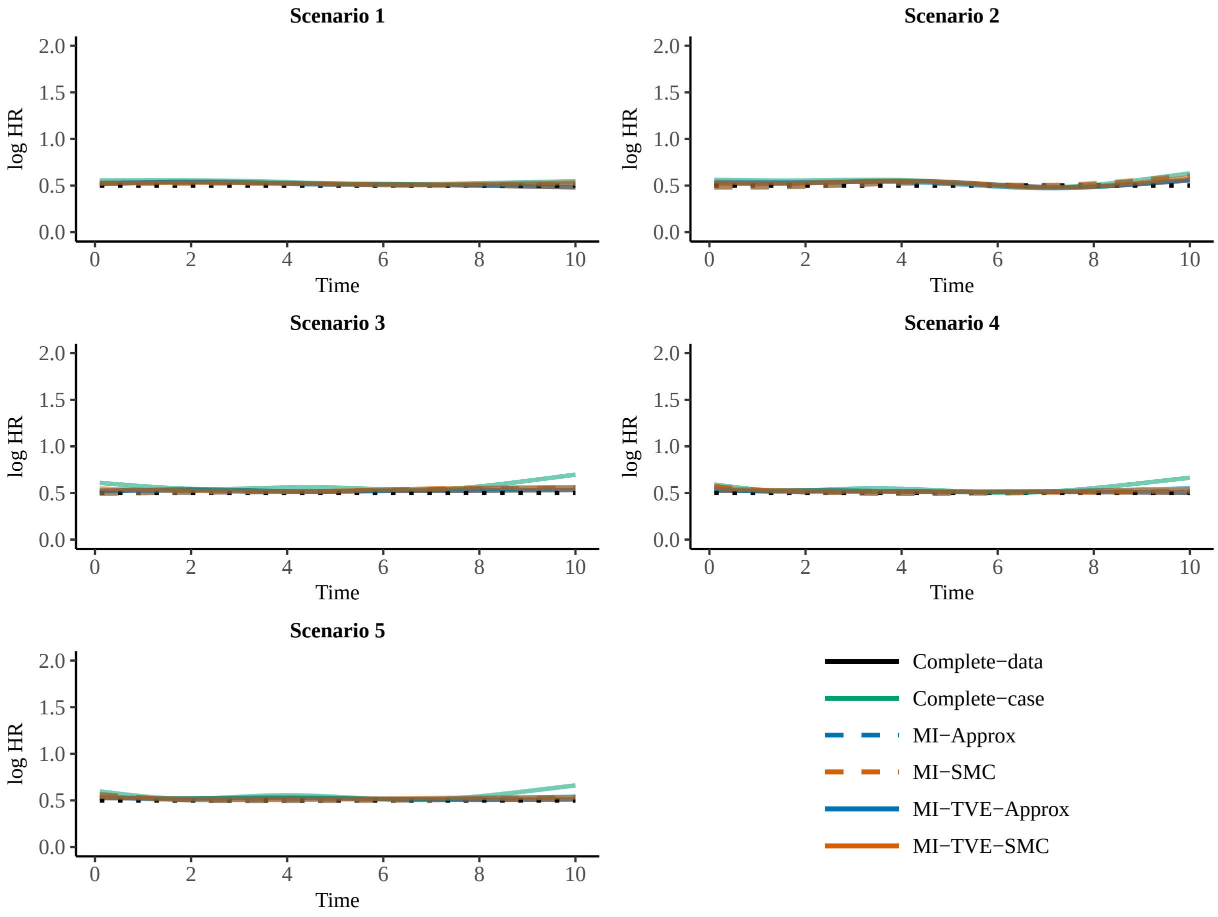

Figure S4. Curve-wise estimates of TVEs for covariate X_2 in the setting with continuous covariates X_1 and X_2. The dotted black line indicates the true curve. The curves are all approximately flat because X_2 always has a non-TVE.

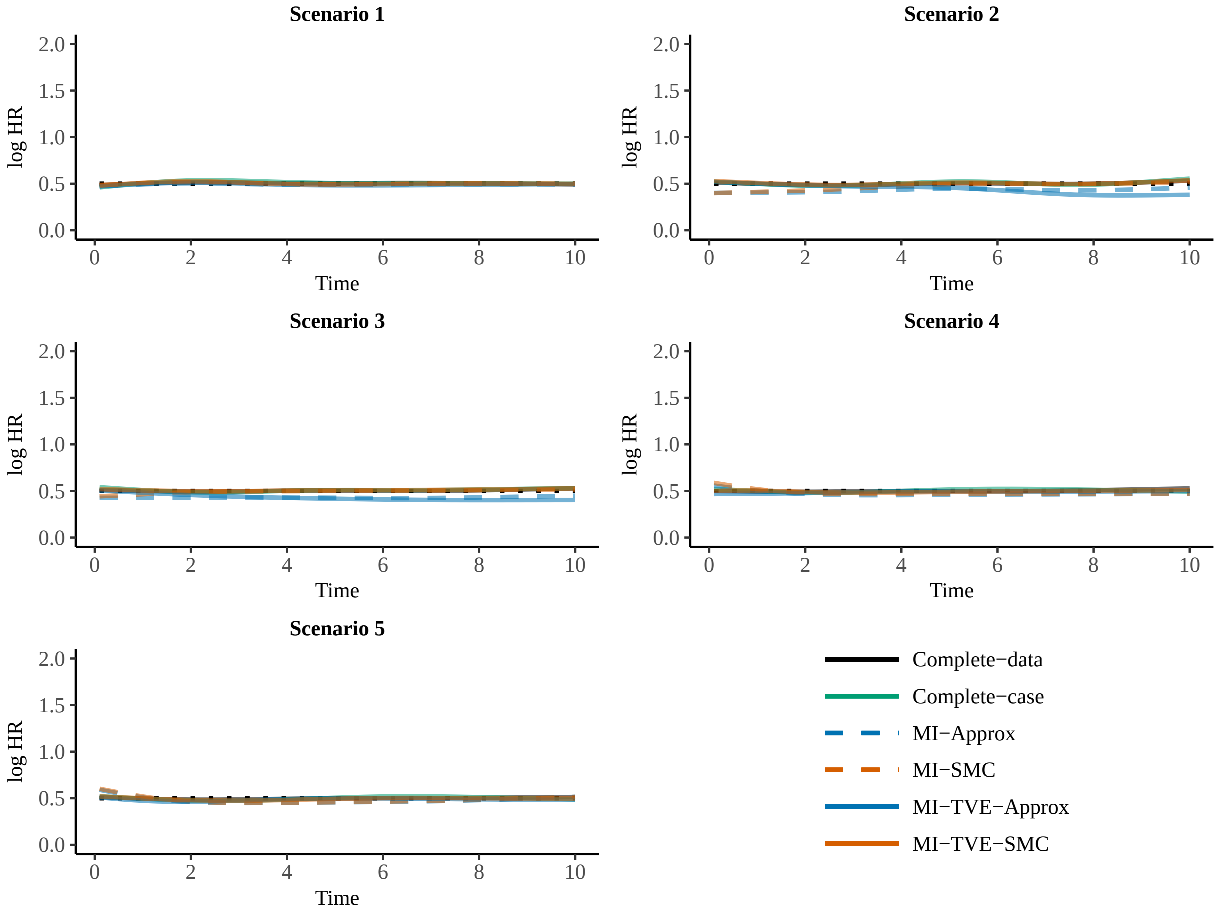

Figure S5. Curve-wise root mean squared error (RMSE) for covariate X_1 in the setting with binary covariates X_1 and X_2.

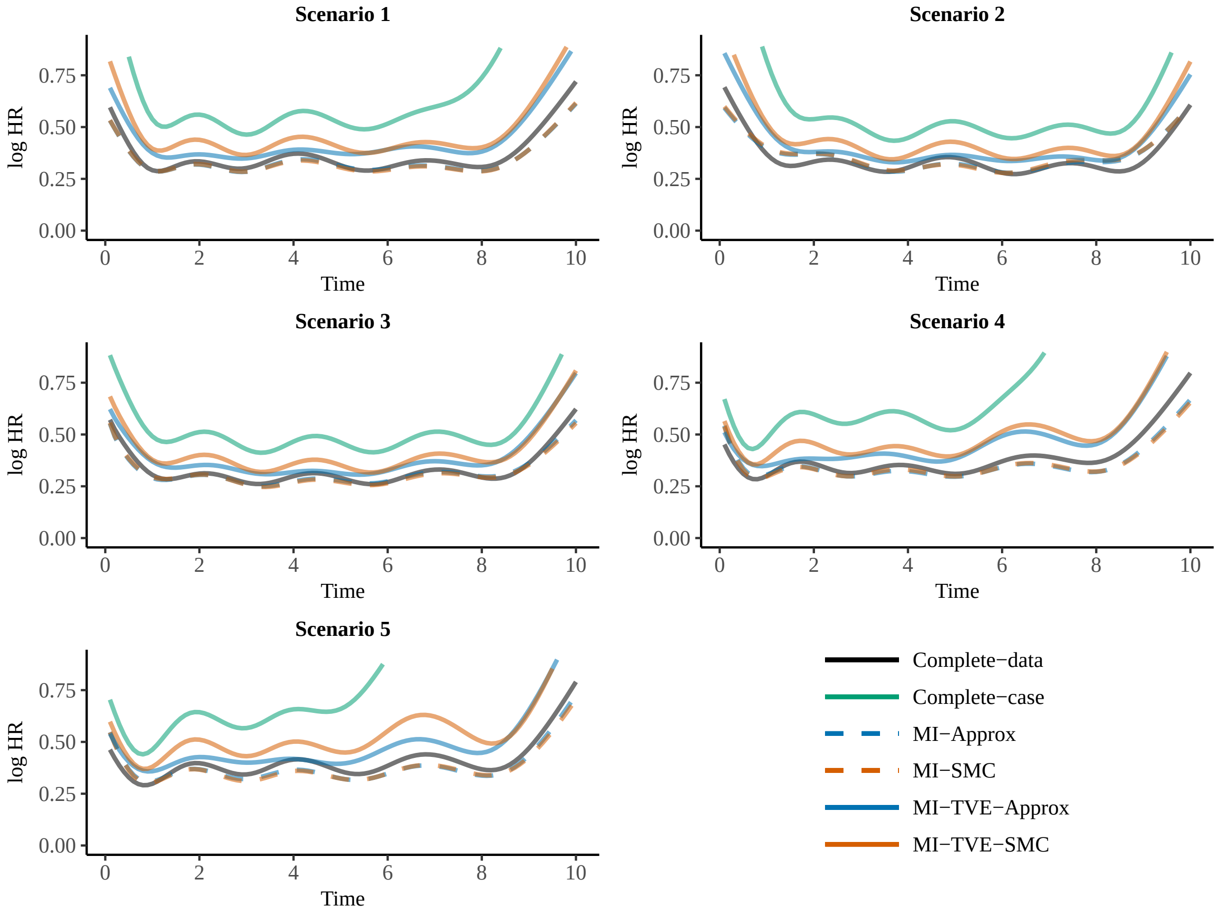

Figure S6. Curve-wise root mean squared error (RMSE) for covariate X_1 in the setting with continuous covariates X_1 and X_2.

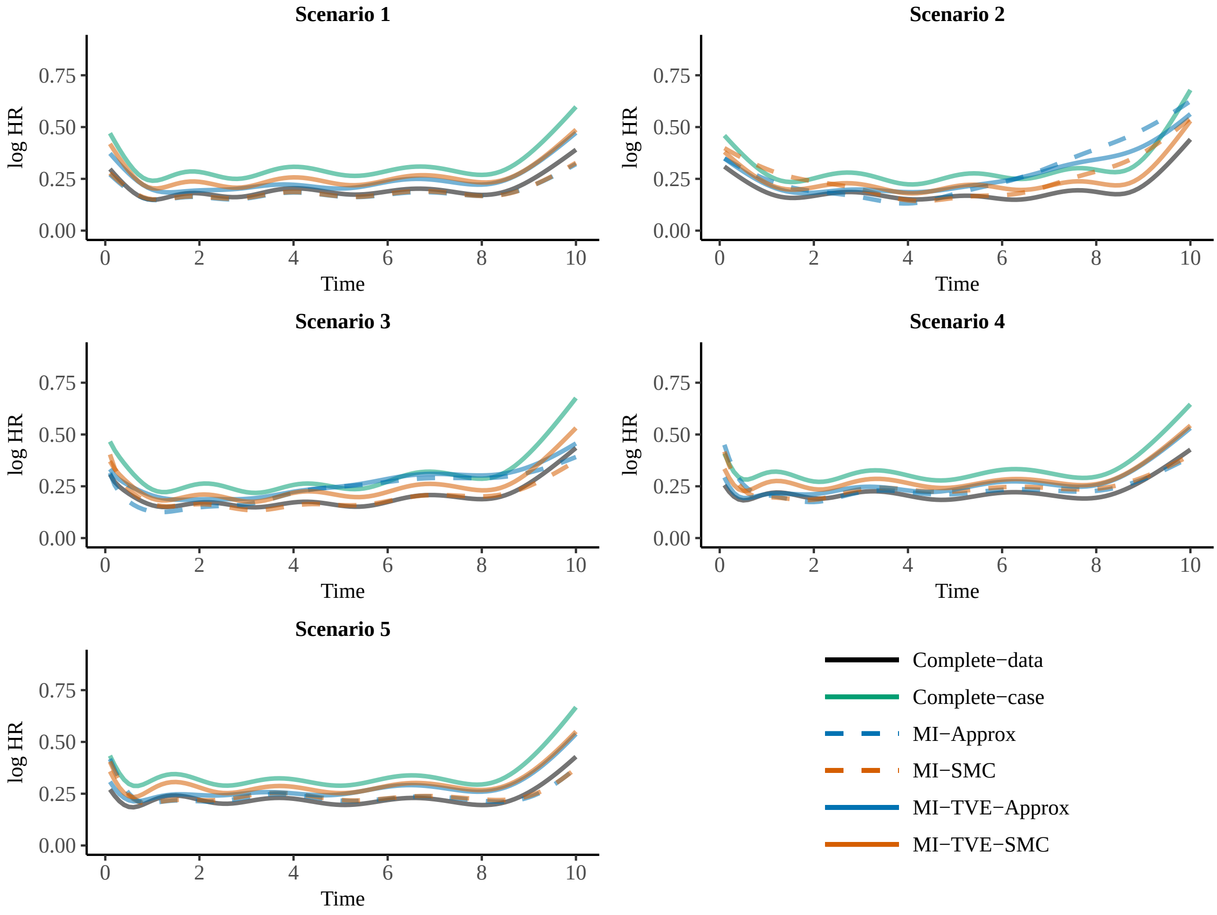

Figure S7. Example estimated TVE curves for covariate X_1 from 100 simulated data sets. Results are shown for the setting with binary covariates X_1 and X_2. The left-hand plots are from the complete data analysis and the right-hand plots are from the MI-TVE-SMC analyses.

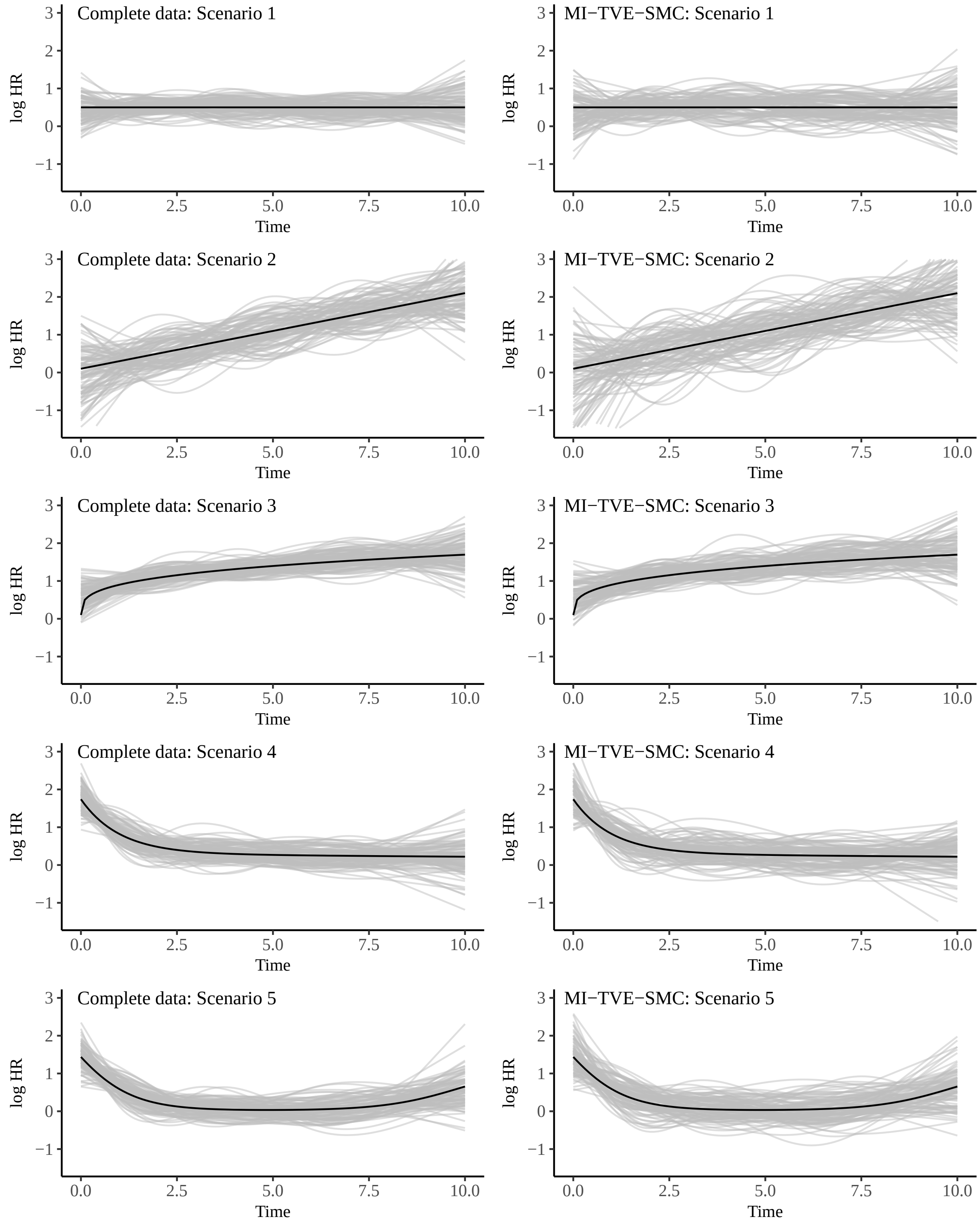

Figure S8. Example estimated TVE curves for covariate X_1 from 100 simulated data sets. Results are shown for the setting with continuous covariates X_1 and X_2. The left-hand plots are from the complete data analysis and the right-hand plots are from the MI-TVE-SMC analyses.

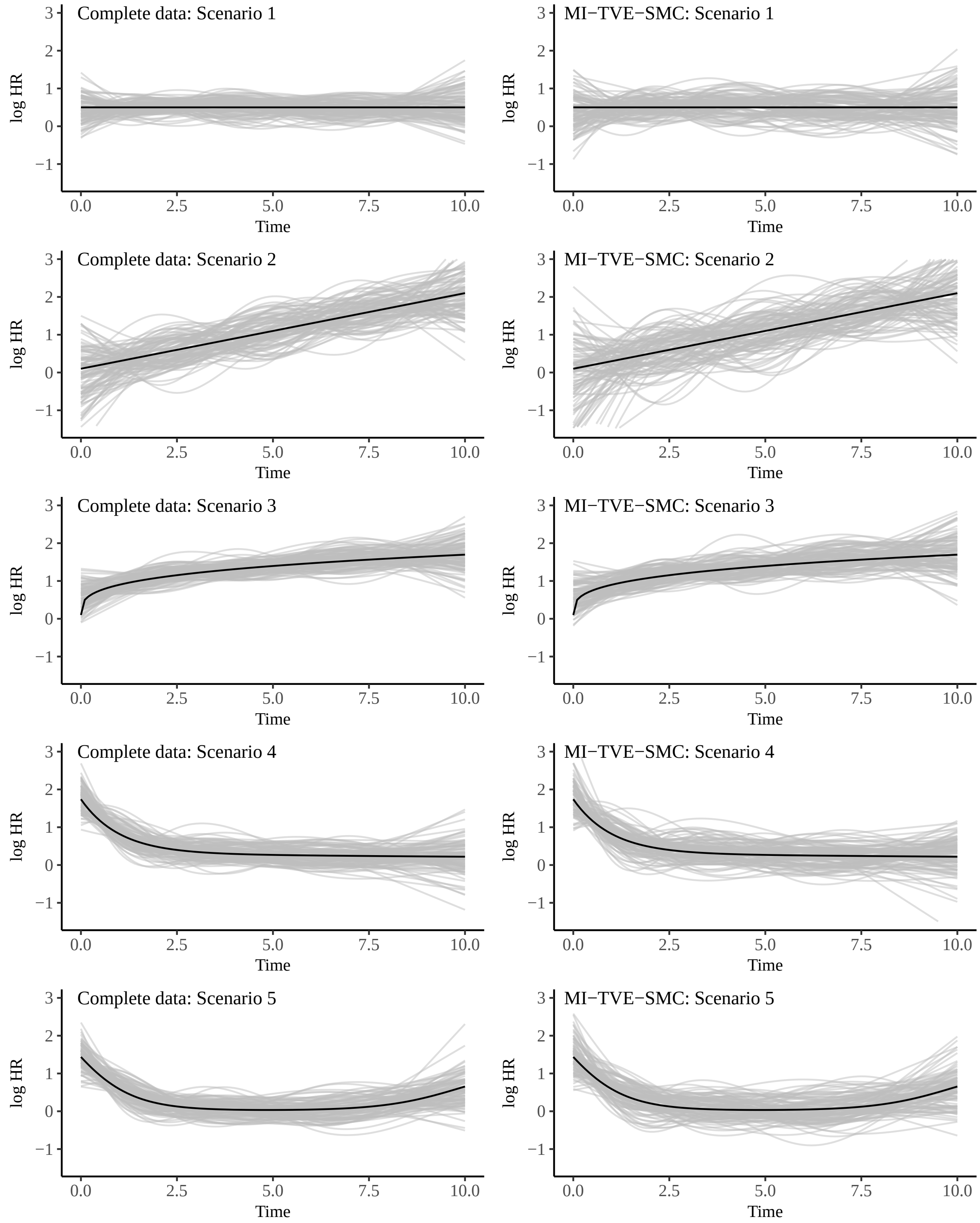

Figure S9. Results from the Rotterdam Breast Cancer Study. Plots showing estimated log hazard ratios as a function of time. The time-varying effects for all covariate were modelled using a restricted cubic spline with 5 knots. Results are shown up to time 10.

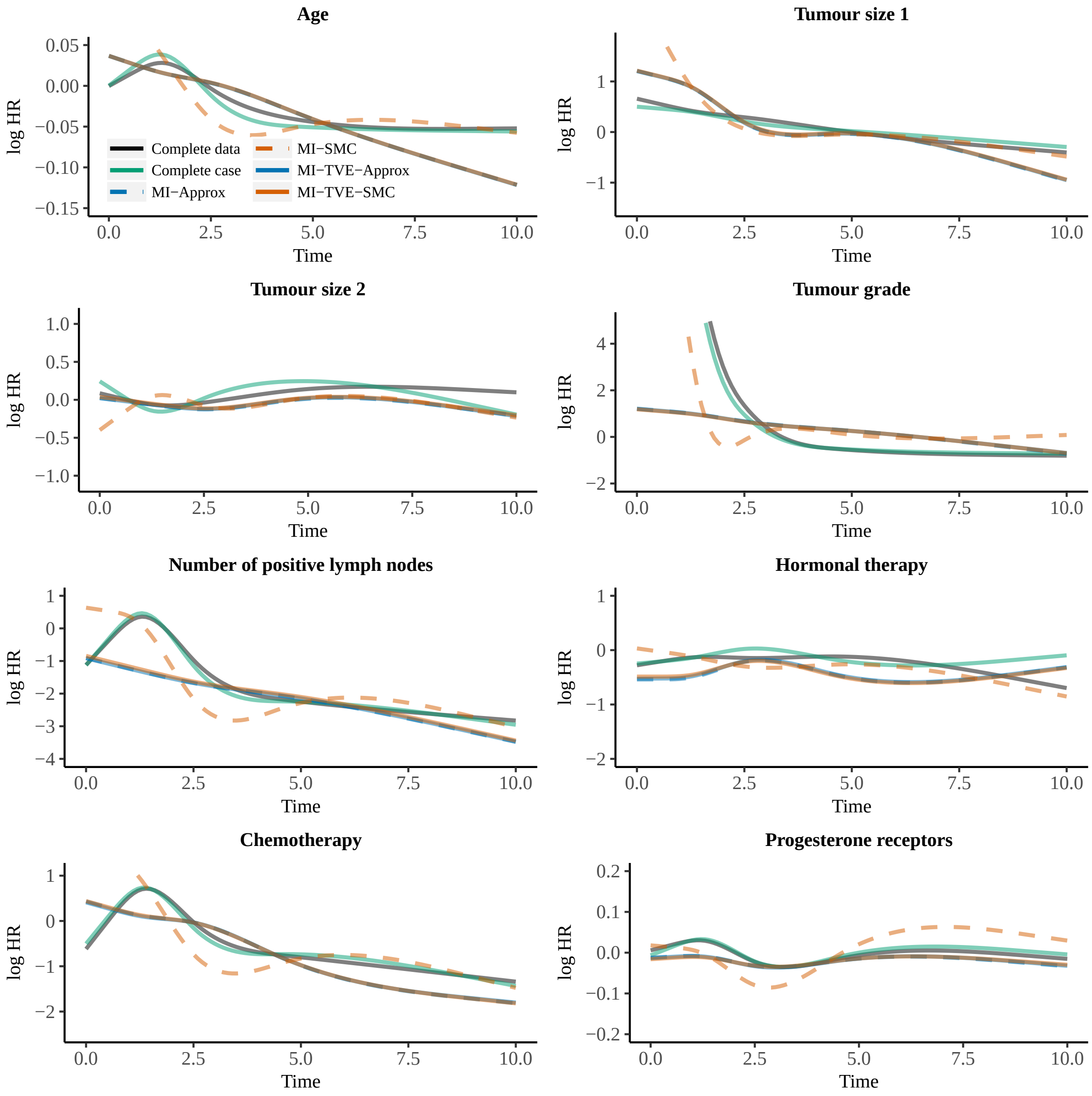

Supplement: Supplementary file 1 — supplementary_materials_revision2.pdf [file SIM-37-3661-s001.pdf]
